# Clinically Applicable Segmentation of Head and Neck Anatomy for Radiotherapy: Deep Learning Algorithm Development and Validation Study

**DOI:** 10.2196/26151

**Published:** 2021-07-12

**Authors:** Stanislav Nikolov, Sam Blackwell, Alexei Zverovitch, Ruheena Mendes, Michelle Livne, Jeffrey De Fauw, Yojan Patel, Clemens Meyer, Harry Askham, Bernadino Romera-Paredes, Christopher Kelly, Alan Karthikesalingam, Carlton Chu, Dawn Carnell, Cheng Boon, Derek D'Souza, Syed Ali Moinuddin, Bethany Garie, Yasmin McQuinlan, Sarah Ireland, Kiarna Hampton, Krystle Fuller, Hugh Montgomery, Geraint Rees, Mustafa Suleyman, Trevor Back, Cían Owen Hughes, Joseph R Ledsam, Olaf Ronneberger

**Affiliations:** 1 DeepMind London United Kingdom; 2 Google Health London United Kingdom; 3 University College London Hospitals NHS Foundation Trust London United Kingdom; 4 Clatterbridge Cancer Centre NHS Foundation Trust Liverpool United Kingdom; 5 University College London London United Kingdom; 6 Google London United Kingdom; 7 Google AI Tokyo Japan

**Keywords:** radiotherapy, segmentation, contouring, machine learning, artificial intelligence, UNet, convolutional neural networks, surface DSC

## Abstract

**Background:**

Over half a million individuals are diagnosed with head and neck cancer each year globally. Radiotherapy is an important curative treatment for this disease, but it requires manual time to delineate radiosensitive organs at risk. This planning process can delay treatment while also introducing interoperator variability, resulting in downstream radiation dose differences. Although auto-segmentation algorithms offer a potentially time-saving solution, the challenges in defining, quantifying, and achieving expert performance remain.

**Objective:**

Adopting a deep learning approach, we aim to demonstrate a 3D U-Net architecture that achieves expert-level performance in delineating 21 distinct head and neck organs at risk commonly segmented in clinical practice.

**Methods:**

The model was trained on a data set of 663 deidentified computed tomography scans acquired in routine clinical practice and with both segmentations taken from clinical practice and segmentations created by experienced radiographers as part of this research, all in accordance with consensus organ at risk definitions.

**Results:**

We demonstrated the model’s clinical applicability by assessing its performance on a test set of 21 computed tomography scans from clinical practice, each with 21 organs at risk segmented by 2 independent experts. We also introduced surface Dice similarity coefficient, a new metric for the comparison of organ delineation, to quantify the deviation between organ at risk surface contours rather than volumes, better reflecting the clinical task of correcting errors in automated organ segmentations. The model’s generalizability was then demonstrated on 2 distinct open-source data sets, reflecting different centers and countries to model training.

**Conclusions:**

Deep learning is an effective and clinically applicable technique for the segmentation of the head and neck anatomy for radiotherapy. With appropriate validation studies and regulatory approvals, this system could improve the efficiency, consistency, and safety of radiotherapy pathways.

## Introduction

### Background

Each year, 550,000 people worldwide are diagnosed with cancer of the head and neck [1]. This incidence is rising [2] and more than doubling in certain subgroups over the last 30 years [3-5]. Where available, most patients will be treated with radiotherapy, which targets the tumor mass and areas at high risk of microscopic tumor spread. However, strategies are needed to mitigate the dose-dependent adverse effects that result from incidental irradiation of normal anatomical structures (*organs at risk*) [6-9].

Thus, the efficacy and safety of head and neck radiotherapy depends on the accurate delineation of organs at risk and tumors, a process known as segmentation or contouring. However, the fact that this process is predominantly done manually means that results may be both inconsistent and imperfectly accurate [10], leading to large inter- and intrapractitioner variability even among experts and thus variation in care quality [11].

**Figure 1:**
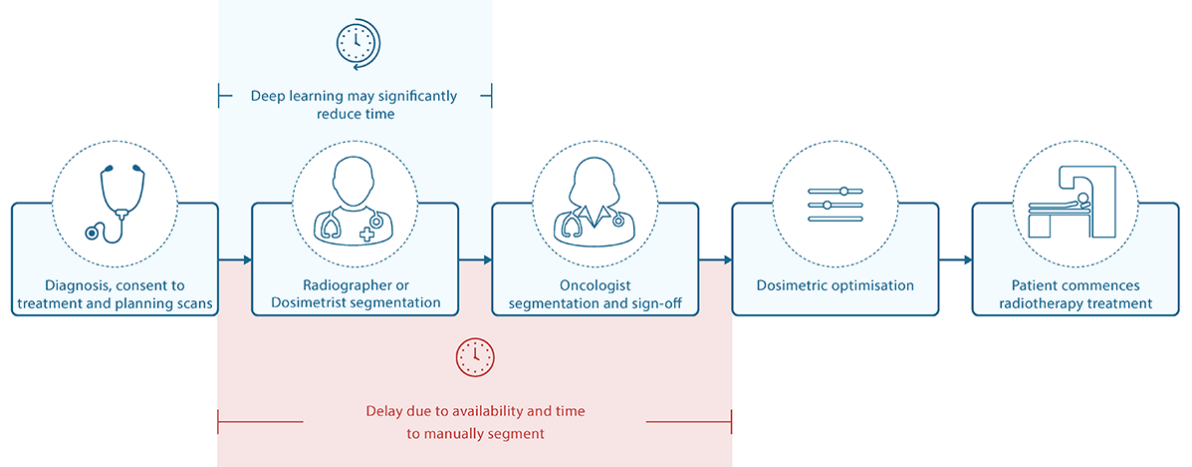
A typical clinical pathway for radiotherapy. After a patient is diagnosed and the decision is made to treat with radiotherapy, a defined workflow aims to provide treatment that is both safe and effective. In the United Kingdom, the time delay between decision to treat and treatment delivery should be no greater than 31 days. Time-intensive manual segmentation and dose optimization steps can introduce delays to treatment.

Segmentation is also very time consuming: an expert can spend 4 hours or more on a single case [12]. The duration of resulting delays in treatment initiation (Figure 1) is associated with an increased risk of both local recurrence and overall mortality [13-15]. Increasing demands for, and shortages of, trained staff already place a heavy burden on health care systems, which can lead to long delays for patients as radiotherapy is planned [16,17], and the continued rise in head and neck cancer incidence may make it impossible to maintain even current temporal reporting standards [4]. Such issues also represent a barrier to *adaptive radiotherapy*—the process of repeated scanning, segmentation, and radiotherapy planning throughout treatment, which maintains the precision of tumor targeting (and organ at risk avoidance) in the face of treatment-related anatomic changes such as tumor shrinkage [18].

Automated (ie, computer-performed) segmentation has the potential to address these challenges. However, most segmentation algorithms in clinical use are atlas based, producing segmentations by fitting previously labeled reference images to the new target scan. This might not sufficiently account for either postsurgical changes or the variability in normal anatomical structures that exist between patients, particularly when considering the variable effect that tumors may have on local anatomy; thus, they may be prone to systematic error. To date, such algorithm-derived segmentations still require significant manual editing, perform at expert levels on only a small number of organs, demonstrate an overall performance in clinical practice inferior to that of human experts, and have failed to significantly improve clinical workflows [19-26].

In recent years, deep learning–based algorithms have proven capable of delivering substantially better performance than traditional segmentation algorithms. Several deep learning–based approaches have been proposed for head and neck cancer segmentation. Some of them use standard convolutional neural network classifiers on patches with tailored pre- and postprocessing [27-31]. However, the U-Net convolutional architecture [32] has shown promise in the area of deep learning–based medical image segmentation [33] and has also been applied to head and neck radiotherapy segmentation [34-47].

Despite the promise that deep learning offers, barriers remain in the application of auto-segmentation in radiotherapy planning. These include the absence of consensus on how *expert* performance is defined, the lack of available methods by which such human performance can be compared with that delivered by automated segmentation processes, and thus how the clinical acceptability of automated processes can be defined.

### Objectives

In this paper, we address these challenges in defining comparison metrics and report a deep learning approach that delineates a wide range of important organs at risk in head and neck cancer radiotherapy scans. We aim to achieve this using a study design that includes (1) the introduction of a clinically meaningful performance metric for segmentation in radiotherapy planning, (2) a representative set of images acquired during routine clinical practice, (3) an unambiguous segmentation protocol for all organs, and (4) a segmentation of each test set image according to these protocols by 2 independent experts. In addition to the model’s generalizability, as demonstrated on two distinct open-source data sets, by achieving performance equal to that of human experts on previously unseen patients from the same hospital site used for training, we aim to demonstrate the clinical applicability of our approach.

## Methods

### Data Sets

University College London Hospitals (UCLH) National Health Service (NHS) Foundation Trust serves an urban, mixed socioeconomic and ethnic population in central London, United Kingdom, and houses a specialist center for cancer treatment. Data were selected from a retrospective cohort of all-adult (aged >18 years) UCLH patients who underwent computed tomography (CT) scans to plan radical radiotherapy treatment for head and neck cancer between January 1, 2008, and March 20, 2016. Both initial CT images and rescans were included in the training data set. Patients with all tumor types, stages, and histological grades were considered for inclusion, as long as their CT scans were available in digital form and were of sufficient diagnostic quality. The standard CT pixel spacing was 0.976×0.976×2.5 mm, and scans with nonstandard spacing (with the exception of 1.25-mm spacing scans that were subsampled) were excluded to ensure consistent performance metrics during training. It should be noted that for the Cancer Imaging Archive (TCIA) test set, the in-plane pixel spacing was not used as an exclusion criterion, *i* ranged from 0.94 to 1.27 mm. For the public domain database for computational anatomy (PDDCA) test set, we included all scans, and the voxels varied between 2 to 3 mm in height and 0.98 to 1.27 mm in axial dimension. Patients’ requests to not have their data shared for research were respected.

Of the 513 patients who underwent radiotherapy at UCLH within the given study dates, a total of 486 patients (94.7%; 838 scans; mean age 57 years; male 337, female 146, and gender unknown 3) met the inclusion criteria. Of note, no scans were excluded because of poor diagnostic quality. Scans from UCLH were split into a training set (389 patients; 663 scans), validation set (51 patients; 100 scans), and test set (46 patients; 75 scans). From the selected test set, 19 patients (21 scans) underwent adjudicated contouring described below. No patient was included in multiple data sets; in cases where multiple scans were present for a single patient, all were included in the same subset. Multiple scans present for a single patient reflect CT scans taken for the purpose of replanning radiotherapy owing to anatomical changes during the course of treatment. It is important for models to perform well in both scenarios as treatment naive and postradiotherapy organ at risk anatomies can differ. However, to avoid potential correlation between the same organs segmented twice in the same data set, care was taken to avoid this in the TCIA test set (described later in this section).

In total, 21 organs at risk were selected throughout the head and neck area to represent a wide range of anatomical regions. We used a combination of segmentations sourced from those used clinically at UCLH and additional segmentations performed in-house by trained radiographers.

We divided our UCLH data set into the following categories: (1) *training set*, used to train the model, a combination of UCLH clinical segmentations and in-house segmentations, some of which were only 2D slices (owing to the time required to segment larger organs manually, we initially relied heavily on sparse segmentations to make efficient use of the radiographers’ time). (2) *UCLH validation set*: used to evaluate model performance and steer additional data set priorities, which used in-house segmentations only, as we did not want to overfit any clinical bias. (3) *UCLH test set*: our primary result set; each scan has every organ at risk labeled and was independently segmented from scratch by 2 radiographers before one of the pairs of scans (chosen arbitrarily) was reviewed and corrected by an experienced radiation oncologist.

As these scans were taken from UCLH patients not present elsewhere, and to consider generalizability, we curated additional open-source CT scans available from The Cancer Genome Atlas Head-Neck Squamous Cell Carcinoma (TCGA-HNSC) and Head-Neck Cetuximab [48-50]. The open-source (category 4) TCIA validation set and (category 5) TCIA test set were both labeled in the same way as our UCLH test set.

**Figure 2:**
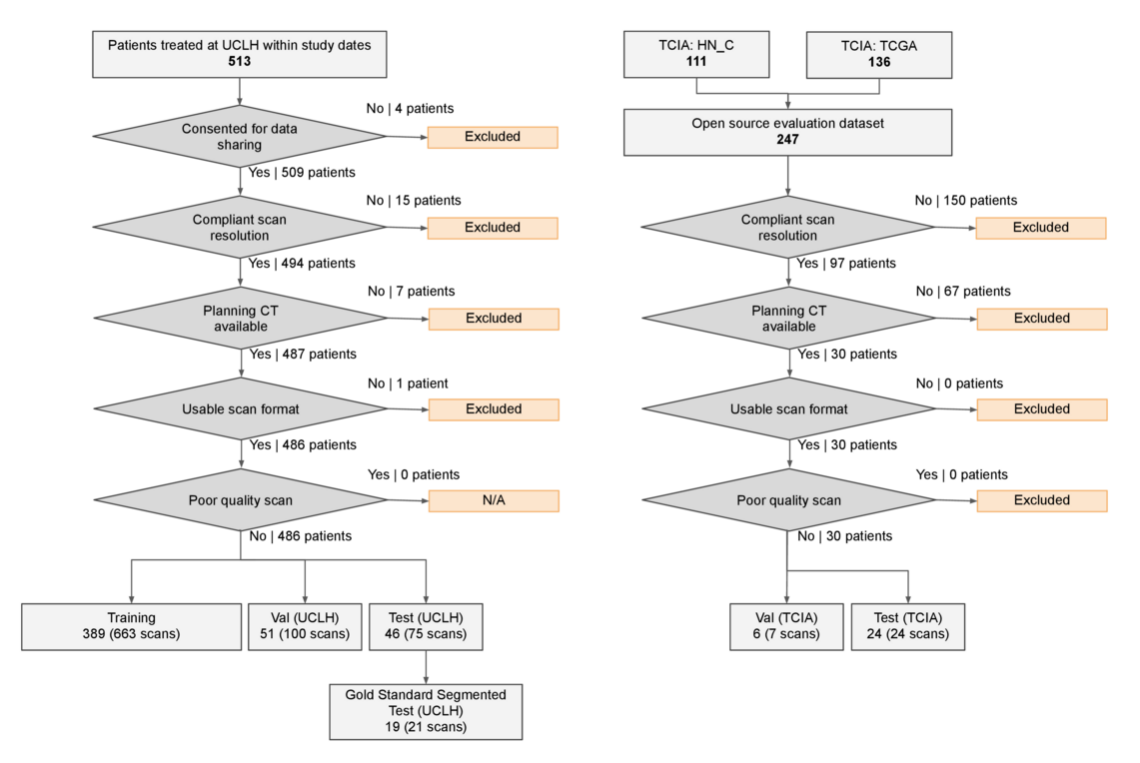
Case selection from the University College London Hospitals and The Cancer Imaging Archive computed tomography data sets. A consort-style diagram demonstrating the application of inclusion and exclusion criteria to select the training, validation, and test sets used in this work. CT: computed tomography; HN_C: Head and Neck Carcinoma; N/A: not applicable; TCIA: The Cancer Imaging Archive; TCGA: The Cancer Genome Atlas; UCLH: University College London Hospitals; Val: validation.

Non-CT planning scans and those that did not meet the same slice thickness as the UCLH scans (2.5 mm) were excluded. These were then manually segmented in-house according to the Brouwer Atlas (the segmentation procedure is described in further detail in the *Clinical Labeling and Annotation* section [51]). We included 31 scans (22 Head-Neck Cetuximab and 9 TCGA-HNSC) that met these criteria, which we further split into validation (6 patients; 7 scans) and test (24 patients; 24 scans) sets (Figure 2). The original segmentations from the Head-Neck Cetuximab data set were not included; a consensus assessment by experienced radiographers and oncologists found the segmentations either nonconformant to the selected segmentation protocol or below the quality that would be acceptable for clinical care. The original inclusion criteria for Head-Neck Cetuximab were patients with stage 3-4 carcinoma of the oropharynx, larynx, and hypopharynx, with a Zubrod performance of 0-1, and meeting predefined blood chemistry criteria between November 2005 and March 2009. The TCGA-HNSC data set included patients treated for head-neck squamous cell carcinoma, with no further restrictions being apparent [48,50].

All test sets were kept separate during model training and validation. Table 1 describes in detail the demographics and characteristics within the data sets; to obtain a balanced demographic in each of the tests, the validation and training data sets, we sampled randomly stratified splits and selected one that minimized the differences between the key demographics in each data set.

In addition, the (6) *PDDCA open-source data set* consisted of 15 patients selected from the Head-Neck Cetuximab open-source data set [48], owing to differences in selection criteria and test, validation, or training set allocation, five scans were present in both the TCIA and PDDCA test sets. This data set was used without further postprocessing and only accessed once to assess the volumetric Dice similarity coefficient (DSC) performance. The PDDCA test set differs from the TCIA test set in both the segmentation protocol and the axial slice thickness. The work by Raudaschl et al [25] provides more details on the data set characteristics and preprocessing.

Table 1 details the characteristics of these data sets and patient demographics.

**Table 1:** Data set characteristics^a^.

Data set			UCLH^b^							TCIA^c^					PDDCA^d^
			Train		Validation		Test		Validation			Test		Test	
Total scans (patients), n			663 (389)		100 (51)		21 (19)		7 (6)			24 (24)		15 (15)	
Average patient age (years)			57.1		57.5		59.6		56.5			59.9		58.6	
**Sex, number of scans (number of patients)**															
	Female	207 (115)		36 (19)		7 (6)		2 (2)			2 (2)		2 (2)		
	Male	450 (271)		64 (32)		14 (13)		5 (4)			20 (20)		9 (9)		
	Unknown	6 (3)		0 (0)		0 (0)		0 (0)			2 (2)		4 (4)		
**Tumor** **site, number of scans (number of patients)**															
	Oropharynx	145 (86)		27 (15)		7 (6)		0 (0)			8 (8)		2 (2)		
	Lip, oral cavity, and pharynx	80 (52)		20 (8)		4 (4)		1 (1)			3 (3)		0 (0)		
	Tongue	53 (26)		8 (5)		1 (1)		2 (2)			7 (7)		0 (0)		
	Larynx	46 (31)		8 (3)		2 (2)		2 (2)			4 (4)		0 (0)		
	Nasopharynx	48 (24)		5 (3)		0 (0)		0 (0)			0 (0)		0 (0)		
	Head, face, and neck	37 (23)		8 (3)		1 (1)		0 (0)			0 (0)		0 (0)		
	Nasal cavity	32 (19)		2 (1)		1 (1)		0 (0)			0 (0)		0 (0)		
	Connective and soft tissue	37 (18)		2 (1)		1 (1)		0 (0)			0 (0)		0 (0)		
	Hypopharynx	17 (10)		1 (1)		0 (0)		2 (1)			1 (1)		0 (0)		
	Accessory sinus	10 (7)		2 (1)		0 (0)		0 (0)			0 (0)		0 (0)		
	Esophagus	6 (2)		1 (1)		0 (0)		0 (0)			0 (0)		0 (0)		
	Other	33 (20)		0 (0)		0 (0)		0 (0)			1 (1)		0 (0)		
	Unknown	119 (71)		16 (9)		4 (3)		0 (0)			0 (0)		13 (13)		
**Source, number of scans (number of patients)**															
	TCGA^e^	—^f^		—		—		2 (2)			7 (7)		0 (0)		
	HN_Cetux^g^	—		—		—		5 (4)			17 (17)		15 (15)		
**Site, number of scans (number of patients)**															
	UCLH	663 (389)		100 (51)		21 (19)		0 (0)			0 (0)		0 (0)		
	MD Anderson Cancer Center	0 (0)		0 (0)		0 (0)		2 (2)			7 (7)		0 (0)		
	Unknown (US)	0 (0)		0 (0)		0 (0)		5 (4)			17 (17)		15 (15)		

^a^Tumor sites were derived from International Classification of Diseases codes. The Cancer Genome Atlas Head-Neck Squamous Cell Carcinoma [52] is an open-source data set hosted on The Cancer Imaging Archive (TCIA). Head-Neck Cetuximab is an open-source data set hosted on TCIA [53]. Public Domain Database for Computational Anatomy data set released as part of the 2015 challenge in the segmentation of head and neck anatomy at the International Conference on Medical Image Computing and Computer Assisted Intervention.

^b^UCLH: University College London Hospitals.

^c^TCIA: The Cancer Imaging Archive.

^d^PDDCA: Public Domain Database for Computational Anatomy.

^e^TCGA: The Cancer Genome Atlas Program.

^f^The University College London Hospitals (UCLH) data set was sourced entirely from UCLH.

^g^HN_Cetux: Head-Neck Cetuximab.

### Clinical Taxonomy

To select the organs at risk to be included in the study, we used the Brouwer Atlas (consensus guidelines for delineating organs at risk for head and neck radiotherapy, defined by an international panel of radiation oncologists [51]). From this, we excluded those regions that required additional magnetic resonance imaging for segmentation, those that were not relevant to routine head and neck radiotherapy, or those that were not used clinically at UCLH. This resulted in a set of 21 organs at risk (Table 2).

**Table 2:** Taxonomy of segmentation regions.

Organ at risk	Total number of labeled slices included	Anatomical landmarks and definition
Brain	11,476	Sits inside the cranium and includes all brain vessels excluding the brainstem and optic chiasm.
Brainstem	34,794	The posterior aspect of the brain including the midbrain, pons, and medulla oblongata. Extending inferior from the lateral ventricles to the tip of the dens at C2. It is structurally continuous with the spinal cord.
Cochlea-left	4526	Embedded in the temporal bone and lateral to the internal auditory meatus.
Cochlea-right	4754	Embedded in the temporal bone and lateral to the internal auditory meatus.
Lacrimal-left	17,186	Concave-shaped gland located at the superolateral aspect of the orbit.
Lacrimal-right	17,788	Concave-shaped gland located at the superolateral aspect of the orbit.
Lens-left	3006	An oval structure that sits within the anterior segment of the orbit. Can be variable in position but never sitting posterior beyond the level of the outer canthus.
Lens-right	3354	An oval structure that sits within the anterior segment of the orbit. Can be variable in position but never sitting posterior beyond the level of the outer canthus.
Lung-left	8340	Encompassed by the thoracic cavity adjacent to the lateral aspect of the mediastinum, extending from the first rib to the diaphragm excluding the carina.
Lung-right	9158	Encompassed by the thoracic cavity adjacent to the lateral aspect of the mediastinum, extending from the first rib to the diaphragm excluding the carina.
Mandible	25,074	The entire mandible bone including the temporomandibular joint, ramus, and body, excluding the teeth. The mandible joins to the inferior aspect of the temporal bone and forms the entire lower jaw.
Optic-nerve-left	3458	A 2 to 5 mm thick nerve that runs from the posterior aspect of the eye, through the optic canal and ends at the lateral aspect of the optic chiasm.
Optic-nerve-right	3012	A 2 to 5 mm thick nerve that runs from the posterior aspect of the eye, through the optic canal and ends at the lateral aspect of the optic chiasm.
Orbit-left	8538	Spherical organ sitting within the orbital cavity. Includes the vitreous humor, retina, cornea, and lens with the optic nerve attached posteriorly.
Orbit-right	8242	Spherical organ sitting within the orbital cavity. Includes the vitreous humor, retina, cornea, and lens with the optic nerve attached posteriorly.
Parotid-left	8984	Multi-lobed salivary gland wrapped around the mandibular ramus. Extends medially to the styloid process and parapharyngeal space. Laterally extending to the subcutaneous fat. Posteriorly extending to the sternocleidomastoid muscle. Anterior extending to posterior border of the mandible bone and masseter muscle. In cases where the retromandibular vein is encapsulated by parotid, this is included in the segmentation.
Parotid-right	11,752	Multi-lobed salivary gland wrapped around the mandibular ramus. Extends medially to the styloid process and parapharyngeal space. Laterally extending to the subcutaneous fat. Posteriorly extending to the sternocleidomastoid muscle. Anterior extending to posterior border of the mandible bone and masseter muscle. In cases where the retromandibular vein is encapsulated by parotid this is included in the segmentation.
Spinal-canal	37,000	Hollow cavity that runs through the foramen of the vertebrae, extending from the base of skull to the end of the sacrum.
Spinal-cord	37,096	Sits inside the spinal canal and extends from the level of the foramen magnum to the bottom of L2.
Submandibular-left	10,652	Sits within the submandibular portion of the anterior triangle of the neck, making up the floor of the mouth and extending both superior and inferior to the posterior aspect of the mandible and is limited laterally by the mandible and medially by the hypoglossal muscle.
Submandibular-right	10,716	Sits within the submandibular portion of the anterior triangle of the neck, making up the floor of the mouth and extending both superior and inferior to the posterior aspect of the mandible and is limited laterally by the mandible and medially by the hypoglossal muscle.

### Clinical Labeling and Annotation

Owing to the large variability of segmentation protocols used and annotation quality in the UCLH data set, all segmentations from all scans selected for inclusion in the training set were manually reviewed by a radiographer with at least 4 years of experience in the segmentation of head and neck organs at risk. Volumes that did not conform to the Brouwer Atlas were excluded from the training. To increase the number of training examples, additional axial slices were randomly selected for further manual organ at risk segmentations to be added based on model performance or perceived imbalances in the data set. These were then produced by a radiographer with at least 4 years of experience in head and neck radiotherapy, arbitrated by a second radiographer with the same level of experience. The total number of examples from the original UCLH segmentations and additional slices are provided in Table 2.

**Figure 3:**
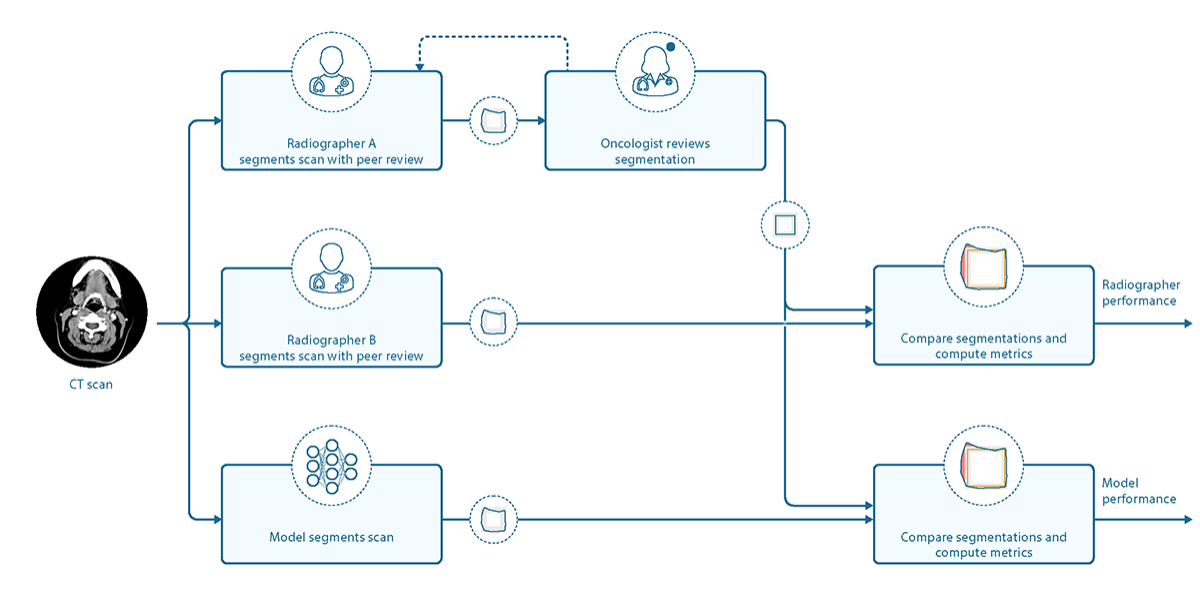
Process for the segmentation of ground truth and radiographer organs at risk volumes. The flowchart illustrates how the ground truth segmentations were created and compared with independent radiographer segmentations and the model. For the ground truth, each computed tomography scan in The Cancer Imaging Archive test set was segmented first by a radiographer and peer reviewed by a second radiographer. This then went through one or more iterations of review and editing with a specialist oncologist before creating a ground truth used to compare with the segmentations produced by both the model and additional radiographer. CT: computed tomography.

For the TCIA test and validation sets, the original dense segmentations were not used owing to poor adherence to the chosen study protocol. To produce the ground truth labels, the full volumes of all 21 organs at risk included in the study were segmented. This was done initially by a radiographer with at least 4 years of experience in the segmentation of head and neck organs at risk and then arbitrated by a second radiographer with similar experience. Further arbitration was then performed by a radiation oncologist with at least 5 years of postcertification experience in head and neck radiotherapy. The same process was repeated with 2 additional radiographers working independently, but after peer arbitration, these segmentations were not reviewed by an oncologist; rather, they became the human reference to which the model was compared. This is schematically shown in Figure 3. Before participation, all radiographers and oncologists were required to study the Brouwer Atlas for head and neck organ at risk segmentation [51] and demonstrate competence in adhering to these guidelines.

### Model Architecture

**Figure 4:**
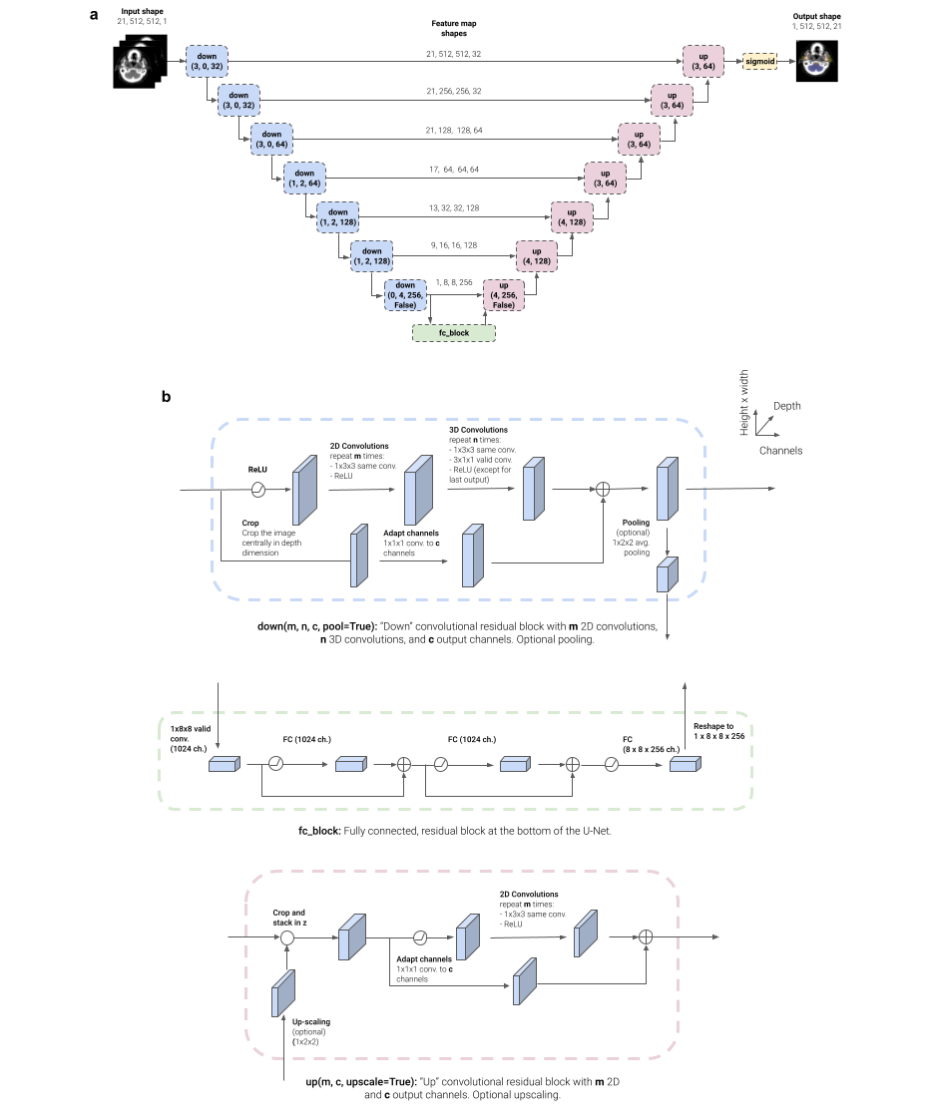
3D U-Net model architecture. (a) At training time, the model receives 21 contiguous computed tomography slices, which are processed through a series of “down” blocks, a fully connected block, and a series of “up” blocks to create a segmentation prediction. (b) A detailed view of the convolutional residual down and up blocks and the residual fully connected block.

We used a residual 3D U-Net architecture with 8 levels (Figure 4). Our network takes in a CT volume (single channel) and outputs a segmentation mask with 21 channels, where each channel contains a binary segmentation mask for a specific organ at risk. The network consists of 7 residual convolutional blocks in the downward path, a residual fully connected block at the bottom, and 7 residual convolutional blocks in the upward path. A 1×1×1 convolution layer with sigmoidal activation produces the final output in the original resolution of the input image. Each predicted slice had 21 slices of context. The 21-slice context (ie, 21 × 2.5 mm=52.5 mm) was found to provide the optimal context. This is not the case with the 21 organs at risk used in this study.

We trained our network with a regularized top-*k*-percent, pixel-wise, binary, cross-entropy loss [54]; for each output channel, the top-*k* loss selects only the *k*% most difficult pixels (those with the highest binary cross-entropy) and only adds their contribution to the total loss. This speeds up training and helps the network to tackle the large class imbalance and to focus on difficult examples.

We regularized the model using standard L2 weight regularization with scale 10^−6^ and extensive data augmentation using random in-plane (ie, in *x* and *y* directions only) translation, rotation, scaling, shearing, mirroring, elastic deformations, and pixel-wise noise. We used uniform translations between −32 and 32 pixels, uniform rotations between −9° and 9°, uniform scaling factors between 0.8° and 1.2°, and uniform shear factors between −0.1 and 0.1. We mirrored the images (and adjusted the corresponding left and right labels) with a probability of 0.5. We performed elastic deformations by placing random displacement vectors (SD 5 mm, in-plane displacements only) on a control point grid with 100×100×100 mm spacing and by deriving the dense deformation field using cubic b-spline interpolation. In the implementation, all spatial transformations are first combined to a dense deformation field, which is then applied to the image using bilinear interpolation and extrapolation with zero padding. We added zero-mean Gaussian intensity noise independently to each pixel with an SD of 20 Hounsfield units.

We trained the model with the Adam optimizer [53] for 120,000 steps and a batch size of 32 (32 graphical processing units) using synchronous stochastic gradient descent. We used an initial learning rate of 10^−4^ and scaled the learning rate by 1/2, 1/8, 1/64, and 1/256 at time steps of 24,000, 60,000, 108,000, and 114,000, respectively.

We used the validation set to select the model that performed at over 95% for most organs at risk according to our chosen surface DSC performance metric, breaking ties by preferring better performance on more clinically impactful organs at risk and the absolute performance obtained.

### Performance Metrics

**Figure 5:**
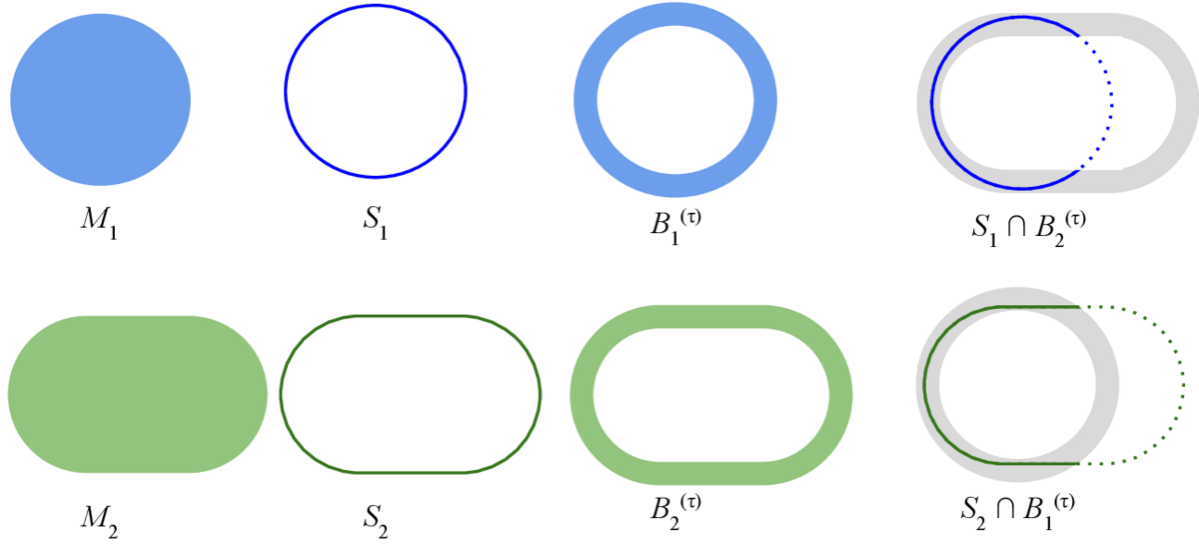
Illustrations of masks, surfaces, border regions, and the “overlapping” surface at tolerance τ.

All performance metrics are reported for each organ independently (eg, separately for just the left parotid), so we only need to deal with binary masks (eg, a left parotid voxel and a non–left-parotid voxel). Masks are defined as a subset of 
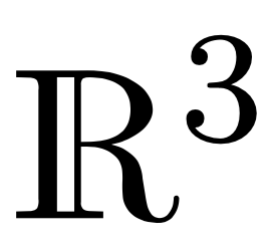
, that is, 
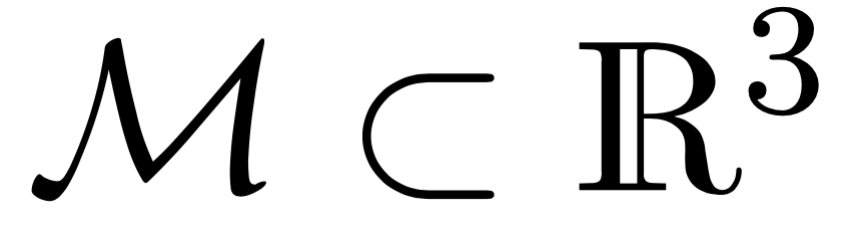
 (Figure 5).

The volume of a mask is denoted as 
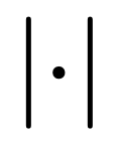
, with


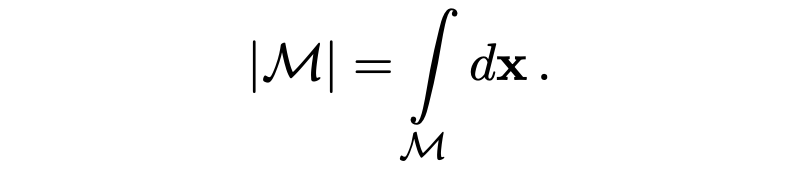


With this notation, the standard (volumetric) DSC for two given masks *M*_1_ and *M*_2_ and can be written as:


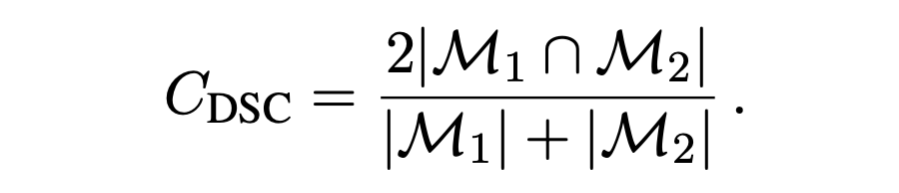


In the case of sparse ground truth segmentations (ie, only a few slices of the CT scan are labeled), we estimate the volumetric DSC by aggregating data from labeled voxels across multiple scans and patients as


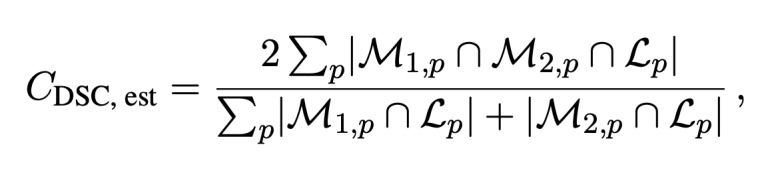


where the mask *M*_1,_*_p_* and the labeled region *L_p_* represent the sparse ground truth segmentation for a patient *p* and the mask *M*_2,_*_p_* is the full volume predicted segmentation for the patient *p*.

Owing to the shortcomings of the volumetric DSC metric for the presented radiotherapy use case, we introduced the *surface DSC* metric, which assesses the overlap of two surfaces (at a specified tolerance) instead of the overlap of two volumes (see *Results* section). A surface is the border of a mask, 
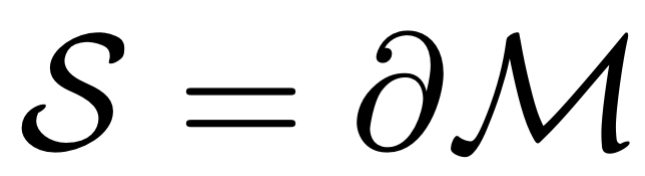
, and the area of the surface is denoted as


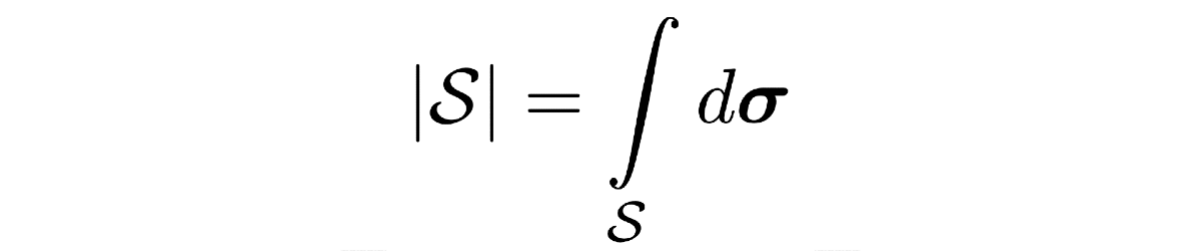


where 
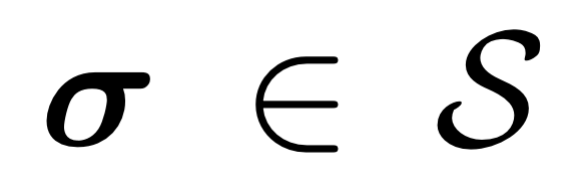
 is a point on the surface using arbitrary parameterization. The mapping from this parameterization to a point in 
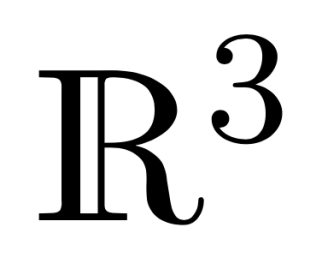
 is denoted as 
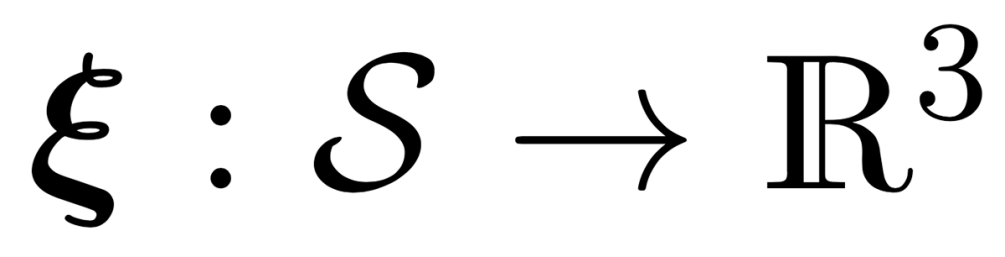
, that is, 
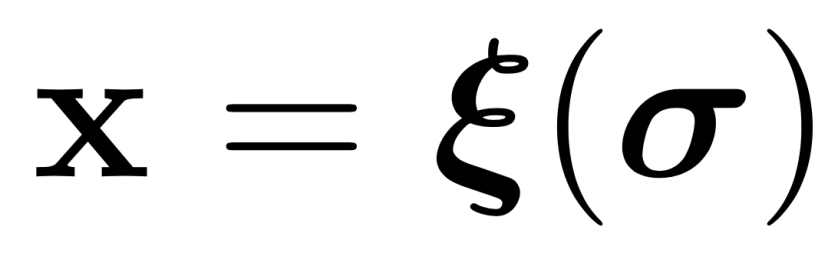
. With this we can define the border region 
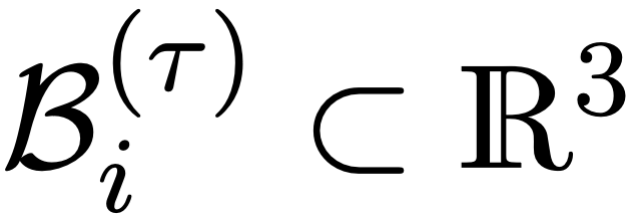
, for the surface *S_i_*, at a given tolerance *τ* as (Figure 5)


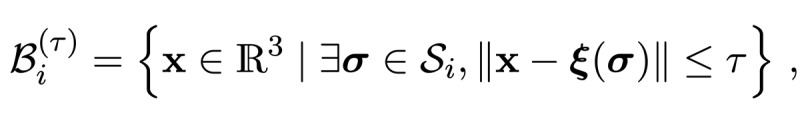


Using these definitions, we can write the *surface DSC at tolerance τ* as


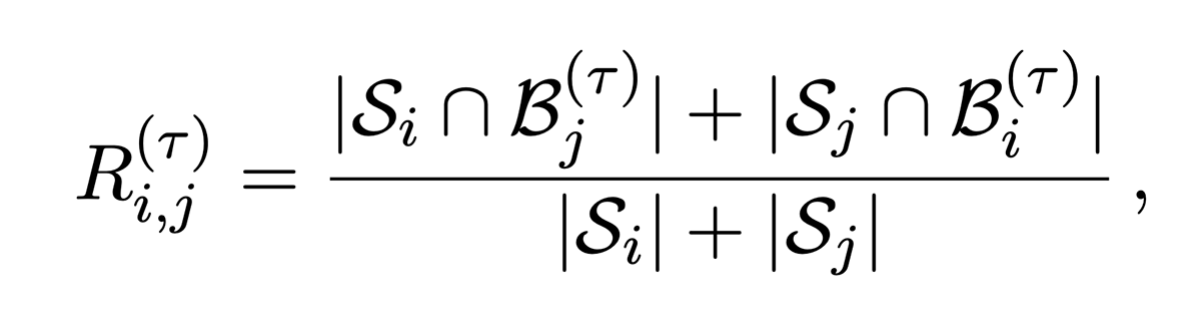


using an informal notation for the intersection of the surface with the boundary, that is,


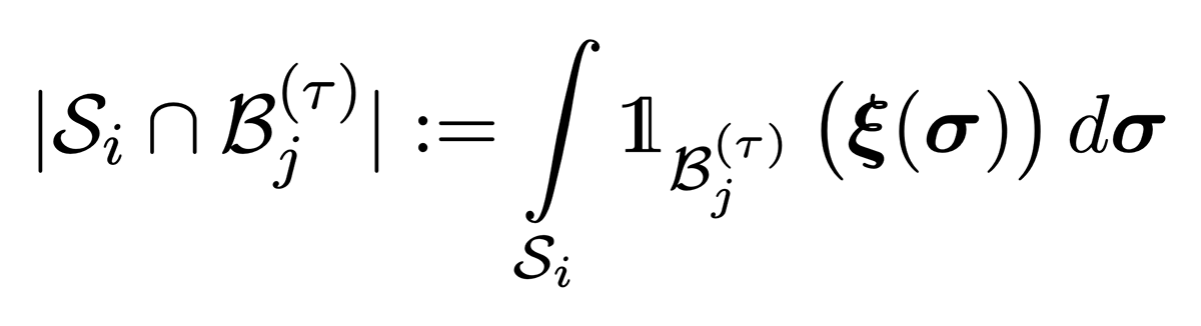


### Implementation of Surface DSC

The computation of surface integrals on sampled images is not straightforward, especially for medical images, where the voxel spacing is usually not equal in all 3 dimensions. The common approximation of the integral by counting the surface voxels can lead to substantial systematic errors.

Another common challenge is the representation of a surface with voxels. As the surface of a binary mask is located between voxels, a definition of *surface voxels* in the raster-space of the image introduces a bias: using foreground voxels to represent the surface leads to an underestimation of the surface, whereas the use of background voxels leads to an overestimation.

**Figure 6:**
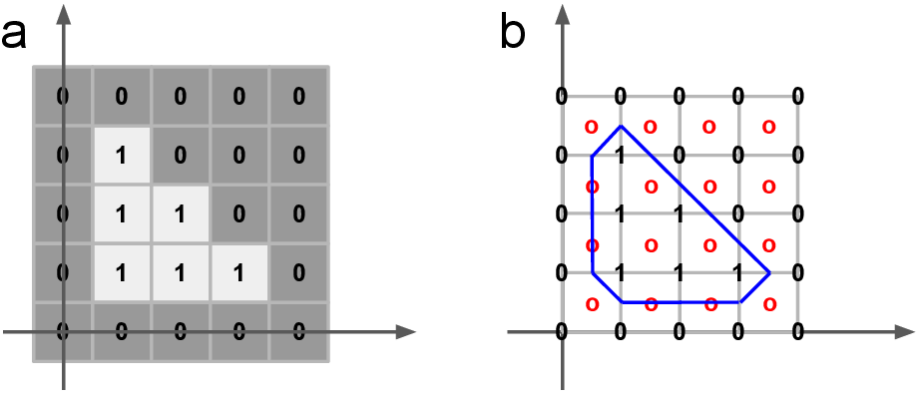
2D illustration of the implementation of the surface Dice similarity coefficient. (a) A binary mask displayed as an image. The origin of the image raster is (0,0). (b) The surface points (red circles) are located in a raster that is shifted half of the raster spacing on each axis. Each surface point has 4 neighbors in 2D (8 neighbors in 3D). The local contour (blue line) assigned to each surface point (red circle) depends on the neighbor constellation.

Our proposed implementation uses a surface representation that provides less-biased estimates but still allows us to compute the performance metrics with linear complexity O(*N*), with *N*: number of voxels). We placed the surface points between the voxels on a raster that is shifted by half of the raster spacing on each axis (see Figure 6 for a 2D illustration).

For 3D images, each point in the raster has 8 neighboring voxels. As we analyzed binary masks, there are only 2^8^=256 possible neighbor constellations. For each of these constellations, we computed the resulting triangles using the marching cube triangulation [55,56] and stored the surface area of the triangles (in mm^2^) in a look-up table. With this look-up table, we then created a surface image (on the above-mentioned raster) that contains zeros at positions that have 8 identical neighbors or the local surface area at all positions that have both foreground and background neighbors. These surface images were created for masks *M*_1_ and *M*_2_. In addition, we created a distance map from each of these surface images using the distance transform algorithm [57]. Iterating over the nonzero elements in the first surface image and looking up the distance from the other surface in the corresponding distance map allows the creation of a list of tuples (surface element area and distance from other surfaces). From this list, we can easily compute the surface area by summing the area of the surface elements that are within the tolerance. To account for the quantized distances, there is only a discrete set 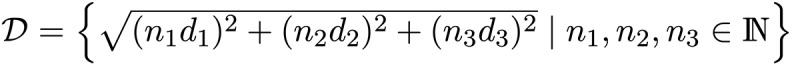 of distances between voxels in a 3D raster with spacing (*d*_1_, *d*_2_, *d*_3_)—we also rounded the tolerance to the nearest neighbor in set *D* for each image before computing the surface DSC. Our open-source implementation of surface DSC provides more details.

## Results

### Selecting Clinically Representative Data Sets

Data sets are described in detail in the Methods section. In brief, the first data set was a representative sample of CT scans used to plan curative-intent radiotherapy of head and neck cancer for patients at UCLH NHS Foundation Trust, a single high-volume center. We performed iterative cycles of model development using the UCLH scans (*training* and *validation* subsets), taking the performance on a previously unseen subset (*test*) as our primary outcome.

It is also important to demonstrate a model’s generalizability to data from previously unseen demographics and distributions. To do this, we curated the test and validation data sets of open-source CT scans. These were collected from the *TCIA test set* [48-50] and the *PDDCA data set* released as part of the 2015 challenge (*PDDCA test set* [25]).

Table 1 details the characteristics of these data sets and their patient demographics. Ethnicity and protected-group status are not reported, as this information was not available in the source systems. In total, 21 organs at risk were selected to represent a wide range of anatomical regions throughout the head and neck. To provide a human clinical comparison for the algorithm, each case was manually segmented by a single radiographer with arbitration by a second radiographer. This was compared with our study’s *gold standard* ground truth graded by 2 other radiographers and arbitrated by one of 2 independent specialist oncologists, each with a minimum of 4 years specialist experience in radiotherapy treatment planning for patients with head and neck cancer.

**Figure 7:**
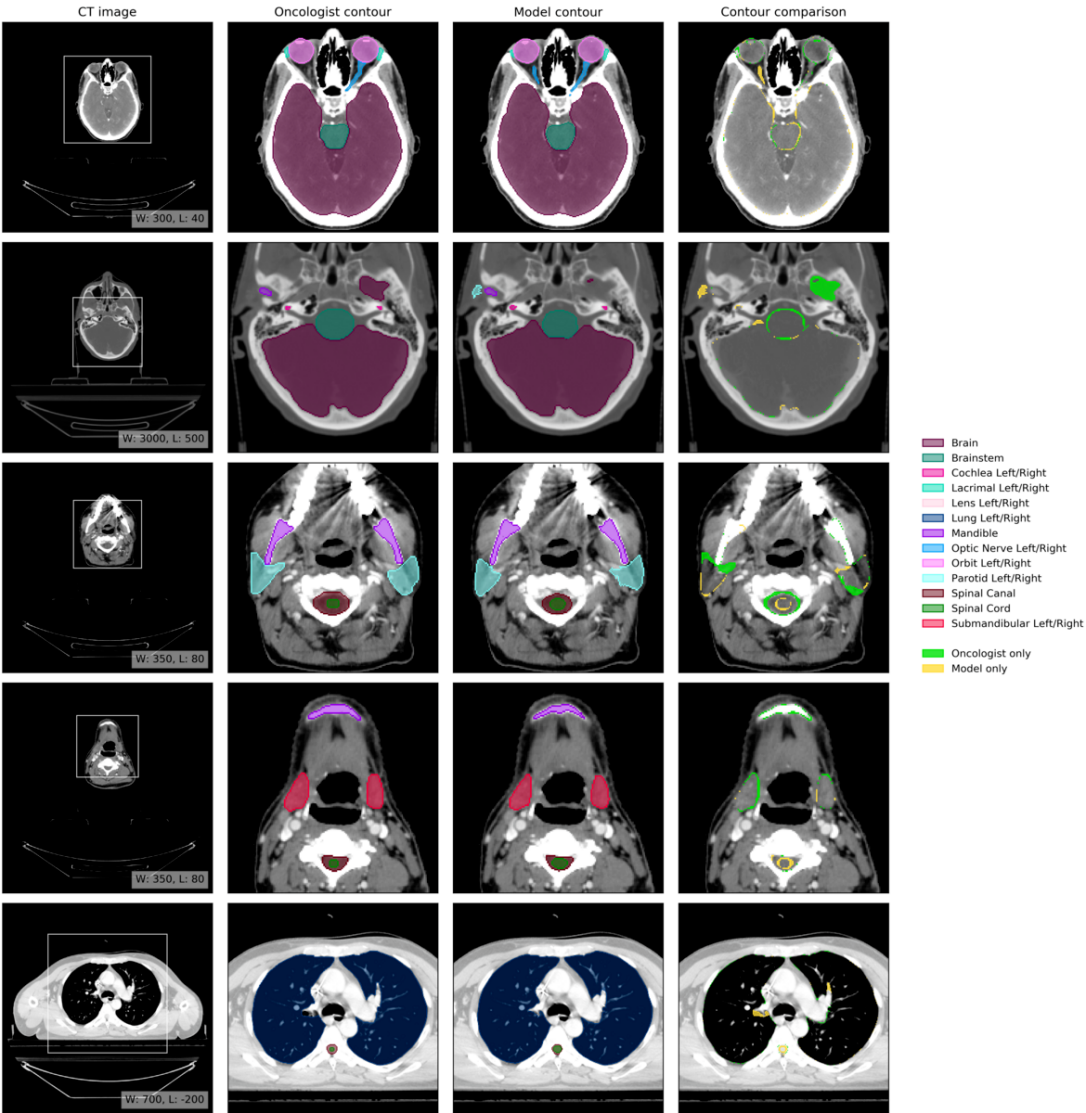
Example results. Computed tomography (CT) image: axial slices at 5 representative levels from the raw CT scan of a male patient aged 55-59 years were selected from the University College London Hospitals data set (patient 20). These were selected to best demonstrate the organs at risks included in the work. The levels shown as 2D slices have been selected to demonstrate all 21 organs at risks included in this study. The window leveling has been adjusted for each to best display the anatomy present. Oncologist contour: the ground truth segmentation, as defined by experienced radiographers and arbitrated by a head and neck specialist oncologist. Model contour: segmentations produced by our model. Contour comparison: contoured by oncologist only (green region) or model only (yellow region). Best viewed on a display. CT: computed tomography.

An example of model performance is shown in Figure 7, two further randomly selected UCLH set scans are shown in Figures S1 and S2 of Multimedia Appendix 1 [19-31,34-46,56-90]. Three randomly selected TCIA set scans are shown in Figures S3, S4 and S5 of Multimedia Appendix 1 to visually demonstrate the model’s generalizability. We compared our performance (model vs oncologist) to radiographer performance (radiographer vs oncologist). For more information on data set selection and inclusion and exclusion criteria for patients and organs at risk, see the *Methods* section.

### A New Metric for Assessing Clinical Performance

In routine clinical care, algorithm-derived segmentation is reviewed and potentially corrected by a human expert, just as those created by radiographers currently are. Segmentation performance is thus best assessed by determining the fraction of the surface that needs to be redrawn. The standard volumetric DSC [91] is not well suited to this because it weighs all regions of misplaced delineation equally and independently of their distance from the surface. For example, two inaccurate segmentations could have a similar volumetric DSC score if one were to deviate from the correct surface boundary by a small amount in many places, whereas the other had a large deviation at a single point. Correcting the former would likely take a considerable amount of time as it would require redrawing almost all of the boundary, whereas the latter could be corrected much faster, potentially with a single edit action.

**Figure 8:**
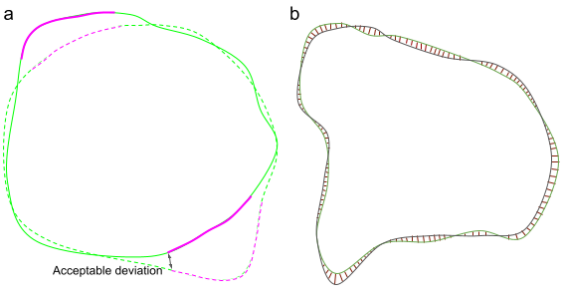
Surface Dice similarity coefficient performance metric. (a) Illustration of the computation of the surface Dice similarity coefficient. Continuous line: predicted surface. Dashed line: ground truth surface. Black arrow: the maximum margin of deviation that may be tolerated without penalty, hereafter referred to by τ. Note that in our use case each organ at risk has an independently calculated value for τ. Green: acceptable surface parts (distance between surfaces ≤τ). Pink: unacceptable regions of the surfaces (distance between surfaces ≤τ). The proposed surface Dice similarity coefficient metric reports the good surface parts compared with the total surface (sum of predicted surface area and ground truth surface area). (b) Illustration of the determination of the organ-specific tolerance. Green: segmentation of an organ by oncologist A. Black: segmentation by oncologist B. Red: distances between the surfaces.

For quantitative analysis, we therefore introduced a new segmentation performance metric, the *surface DSC* (Figure 8), which assesses the overlap of two surfaces (at a specified tolerance) instead of the overlap of two volumes. This provides a measure of agreement between the surfaces of two structures, which is where most of the human effort in correcting is usually expended. In doing so, we also addressed the volumetric DSC’s bias toward large organs at risk, where the large (and mostly trivial) internal volume accounts for a much larger proportion of the score.

When evaluating the surface DSC, we must define a threshold within which the variation is clinically acceptable. To do this, we first defined the organ-specific tolerances (in mm) as a parameter of the proposed metric, τ. We computed these acceptable tolerances for each organ by measuring the interobserver variation in segmentations between 3 different consultant oncologists (each with over 10 years of experience in organ at risk delineation) on the validation subset of TCIA images.

To penalize both false-negative and false-positive parts of the predicted surface, our proposed metrics measure both the nonsymmetric distances between the surfaces and then normalize them by the combined surface area. Similar to volumetric DSC, the surface DSC ranges from 0 (no overlap) to 1 (perfect overlap).

This means that approximately 95% of the surface was properly outlined (ie, within τ mm of the correct boundary), whereas 5% needs to be corrected. There is no consensus as to what constitutes a nonsignificant variation in such a segmentation. Thus, we selected a surface DSC of 0.95, a stringency that likely far exceeds the expert oncologist intrarater concordance [19,92]. For a more formal definition and implementation, see the *Methods* section.

### Model Performance

Model performance was evaluated alongside that of therapeutic radiographers (each with at least 4 years of experience) segmenting the test set of UCLH images independently of the oncologist-reviewed scans (which we used as our ground truth).

**Figure 9:**
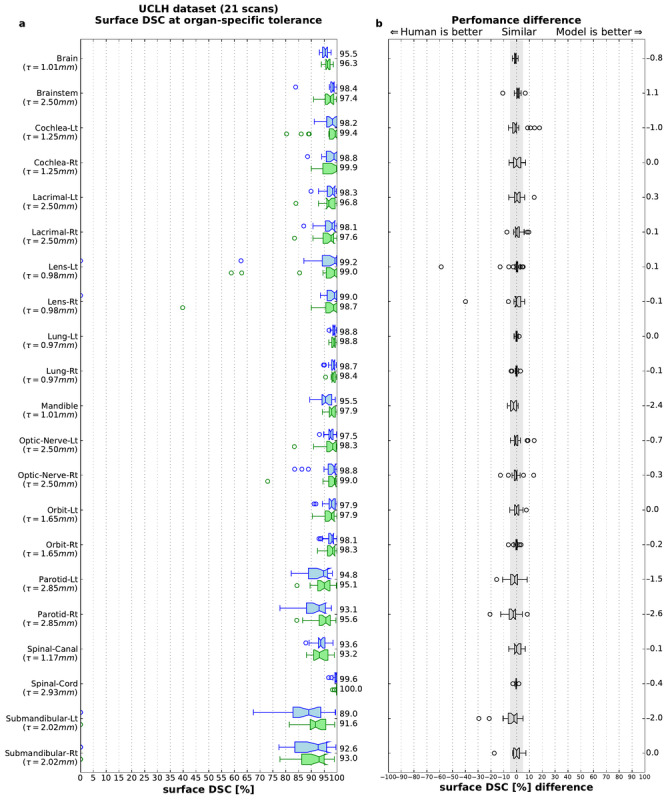
University College London Hospitals (UCLH) test set: quantitative performance of the model in comparison with radiographers. (a) The model achieves a surface Dice similarity coefficient similar to humans in all 21 organs at risk (on the UCLH held out test set) when compared with the gold standard for each organ at an organ-specific tolerance τ. Blue: our model; green: radiographers. (b) Performance difference between the model and the radiographers. Each blue dot represents a model-radiographer pair. The gray area highlights nonsubstantial differences (−5% to +5%). The box extends from the lower to upper quartile values of the data, with a line at the median. The whiskers indicate most extreme, nonoutlier data points. Where data lie outside, an IQR of 1.5 is represented as a circular flier. The notches represent the 95% CI around the median. DSC: Dice similarity coefficient; UCLH: University College London Hospitals.

The model performed similarly to humans. For all organs at risk studied, there was no clinically meaningful difference between the deep learning model’s segmentations and those of the radiographers (Figure 9 and Tables S1 and S2, Multimedia Appendix 1). For details on the number of labelled scans in the UCLH test set, see Table S3 in Multimedia Appendix 1.

**Figure 10:**
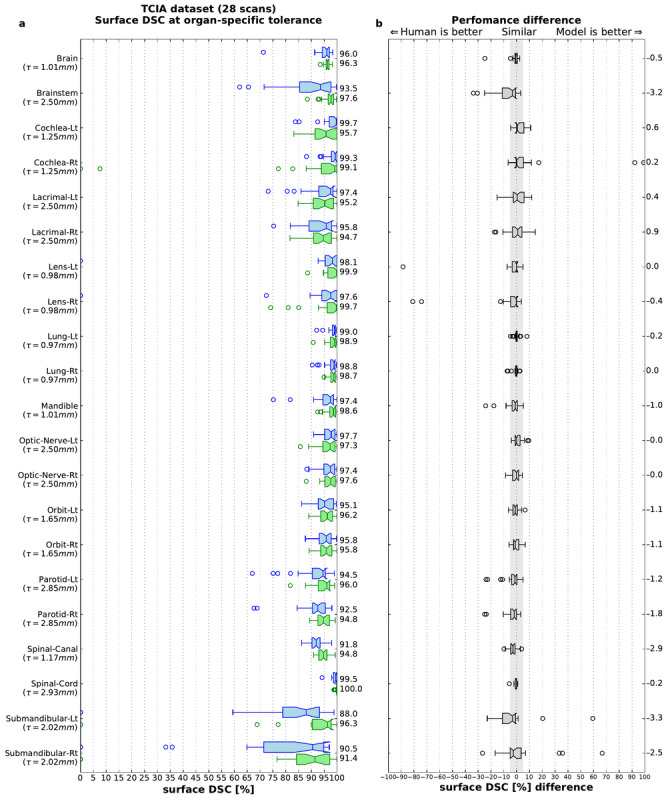
Model generalizability to an independent test set from The Cancer Imaging Archive (TCIA). Quantitative performance of the model on TCIA test set in comparison with radiographers. (a) Surface Dice similarity coefficient (on the TCIA open-source test set) for the segmentations compared with the gold standard for each organ at an organ-specific tolerance τ. Blue: our model, green: radiographers. (b) Performance difference between the model and the radiographers. Each blue dot represents a model-radiographer pair. Red lines show the mean difference. The gray area highlights nonsubstantial differences (−5% to +5%). The box extends from the lower to upper quartile values of the data, with a line at the median. The whiskers indicate most extreme, nonoutlier data points. Where data lie outside, an IQR of 1.5 is represented as a circular flier. The notches represent the 95% CI around the median. DSC: Dice similarity coefficient; TCIA: The Cancer Imaging Archive.

To investigate the generalizability of our model, we additionally evaluated the performance of open-source scans (*TCIA test set*). These were collected from sites in the United States, where patient demographics, clinical pathways for radiotherapy, and scanner type and parameters differed from our UK training set in meaningful ways. Nevertheless, model performance was preserved, and in 90% (19/21) organs at risk, the model was performed within the threshold defined for human variability (Figure 10). The fact that performance in 2 organs at risk (brainstem and right lens) was less than that in UK data may relate to issues of image quality in several TCIA test set scans.

For more detailed results demonstrating surface DSC and volumetric DSC for each individual patient from the TCIA test set, see Table S4 and Table S5, respectively, in Multimedia Appendix 1.

### Comparison With Previous Work

An accurate quantitative comparison with previously published literature is difficult because of inherent differences in definitions of ground truth segmentations and varied processes of arbitration and consensus building. Given that the use of surface DSC is novel in this study, we also reported the standard volumetric DSC scores achieved by our algorithm (despite the shortcomings of this method) so that our results can be directly compared with those in the existing literature. An overview of past papers that have reported mean volumetric DSC for unedited automatic delineation of head and neck CT organs at risk can be found in Table S6, Multimedia Appendix 1. Each used different data sets, scanning parameters, and labeling protocols, meaning that the resulting volumetric DSC results varied significantly. No study, other than ours, segmented the lacrimal glands. We compared these results with those obtained when we applied our model to three different data sets: the TCIA open-source test set, an additional test set from the original UCLH data set (*UCLH test set*) and the data set released by the PDDCA as part of the 2015 Medical Image Computing and Computer Assisted Intervention head and neck radiotherapy organ at risk segmentation challenge (*PDDCA test set* [25]). To contextualize the performance of our model, radiographer performance is shown on the TCIA test set, and oncologist interobserver variation is shown on the UCLH test set.

Although not the primary test set, we nevertheless present per-patient surface DSC and volumetric DSC for the PDDCA test set in Table S7 and Table S8 in Multimedia Appendix 1, respectively.

## Discussion

### Principal Findings

We demonstrated an automated deep learning–based segmentation algorithm that can perform as well as experienced radiographers for head and neck radiotherapy planning. Our model was developed using CT scans derived from routine clinical practice and therefore should be applicable in a hospital setting for the segmentation of organs at risk, routine radiation therapy quality assurance peer review, and in reducing the associated variability between different specialists and radiotherapy centers [93].

Clinical applicability must be supported not only by high model performance but also by evidence of model generalizability to new external data sets. To achieve this, we presented these results on three separate test sets, one of which (the PDDCA test set) uses a different segmentation protocol. In this study, performance in most organs at risk was maintained when tested on scans taken from a range of previously unseen international sites. Although these scans varied in patient demographics, scanning protocol, device manufacturer, and image quality, the model still achieved human performance on 19 of the 21 organs at risk studied; only the right lens and brainstem were below radiographer performance. For these organs at risk, the performance of the model might have been lower than expert performance owing to lower image quality. This is particularly evident for the right lens, where the anatomical borders were quite indistinct in some TCIA test set cases, thus preventing full segmentation by the model (Figure S6, Multimedia Appendix 1). Moreover, a precise CT definition of the brainstem’s proximal and distal boundaries is lacking, a factor that might have contributed to labeling variability and thus to decreased model performance. Finally, demographic bias may have resulted from the TCIA data set selection for cases of more advanced head and neck cancer [48] or from variability in the training data [10].

One major contribution of this paper is the presentation of a performance measure that represents the clinical task of organ at risk correction. In the first preprint of this work, we introduced surface DSC [70], a metric conceived to be sensitive to clinically significant errors in organ at risk delineation. Surface DSC has recently been shown to be more strongly correlated with the amount of time required to correct segmentation for clinical use than traditional metrics, including volumetric DSC [94,95]. Small deviations in organ at risk border placement can have a potentially serious impact, increasing the risk of debilitating side effects for the patient. Misplacement by only a small offset may thus require the entire region to be redrawn, and in such cases, an automated segmentation algorithm may offer no time savings. Volumetric DSC is relatively insensitive to such small changes in large organs, as the absolute overlap is also large. Difficulties identifying the exact borders of smaller organs can result in large differences in volumetric DSC, even if these differences are not clinically relevant in terms of their effect on radiotherapy treatment. By strongly penalizing border placement outside a tolerance determined by consultant oncologists, the surface DSC metric resolves these issues.

Although volumetric DSC is therefore not representative of clinical consequences, it remains to be the most popular metric for evaluating segmentation models and therefore the only metric that allows comparison with previously published works. In recent years, fully convolutional networks have become the most popular and successful methodology for organ at risk segmentation in head and neck CT for de novo radiotherapy planning [40-45,58-69]. Although not directly comparable owing to different data sets and labeling protocols, our volumetric DSC results compare favorably with the existing published literature for many of the organs at risk (see Table S6 and Figure S7, Multimedia Appendix 1, for more details on this and other prior publications). In organs at risk with inferior volumetric DSC scores compared with the published literature, both our model and human radiographers achieved similar scores. This suggests that current and previously published results are difficult to compare, either because of the inclusion of more difficult cases than previous studies or because of different segmentation and scanning protocols. To allow more objective comparisons of different segmentation methods, we made our labeled TCIA data sets freely available to the academic community (see the Acknowledgments section on data availability). At least 11 auto-segmentation software solutions are currently available commercially, with varying claims regarding their potential to lower segmentation time during radiotherapy planning [96]. The principal factor that determines whether automatic segmentation is time saving during the radiotherapy workflow is the degree to which automated segmentations require correction by oncologists.

The wide variability in state-of-the-art and limited uptake in routine clinical practice motivates the need for clinical studies evaluating model performance in practice. Future work will seek to define the clinical acceptability of the segmented organs at risk produced by our models and estimate the time saving that could be achieved during the radiotherapy planning workflow in a real-world setting.

A number of other study limitations should be addressed in future studies. First, we included only planning CT scans because magnetic resonance imaging and positron emission tomography scans were not routinely performed for all patients in the UCLH data set. Some organ at risk classes, such as optic chiasm, require co-registration with MR images for optimal delineation, and access to additional imaging has been shown to improve the delineation of optic nerves [29]. As a result, certain organ at risk classes were deliberately excluded from this CT-based project and will be addressed in future work that will incorporate magnetic resonance imaging scans. A second limitation is with regard to the classes of organs at risk in this study. Although we presented one of the largest sets of reported organs at risk in the literature [44,97,98], some omissions occurred (eg, oral cavity) owing to an insufficient number of examples in the training data that conformed to a standard international protocol. The number of oncologists used in the creation of our ground truth may not have fully captured the variability in organ at risk segmentation or may have been biased toward a particular interpretation of the Brouwer Atlas used as our segmentation protocol. Even in an organ as simple as the spinal cord that is traditionally reliably outlined by auto-segmentation algorithms, there is ambiguity between the inclusion of, for example, the nerve roots. Such variation may widen the thresholds of acceptable deviation in favor of the model, despite a consistent protocol. Future studies will address these deficits alongside time-consuming lymph node segmentation.

Finally, neither of the test sets used in this study included the patients’ protected-characteristic status. This is a significant limitation, as it prevents the study of intersectional fairness.

### Conclusions

In conclusion, we demonstrated that deep learning can achieve human expert–level performance in the segmentation of head and neck organs at risk in radiotherapy planning CT scans, using a clinically applicable performance metric designed for this clinical scenario. We provided evidence of the generalizability of this model by testing it on patients from different geographies, demographics, and scanning protocols. This segmentation algorithm was performed with similar accuracy compared with experts and has the potential to improve the speed, efficiency, and consistency of radiotherapy workflows, with an expected positive influence on patient outcomes. Future work will investigate the impact of our segmentation algorithm in clinical practice.

## Appendix

Multimedia Appendix 1 Additional Tables S1-S8 and Figures S1-S7 show further visual examples of model outputs, performance metrics and detailed comparisons to previously published works.

## Abbreviations

CT: computed tomography
DSC: Dice similarity coefficient
NHS: National Health Service
PDDCA: public domain database for computational anatomy
TCGA-HNSC: The Cancer Genome Atlas Head-Neck Squamous Cell Carcinoma
TCIA: The Cancer Imaging Archive
UCLH: University College London Hospitals
