# Supplementary material for: Clinically Applicable Segmentation of Head and Neck Anatomy for Radiotherapy: Deep Learning Algorithm Development and Validation Study
Source: J Med Internet Res. 2021 Jul 12;23(7):e26151. doi: 10.2196/26151 (PMC8314151; doi:10.2196/26151)
Supplement: Multimedia Appendix 1 [file jmir_v23i7e26151_app1.pdf]

A. Appendix

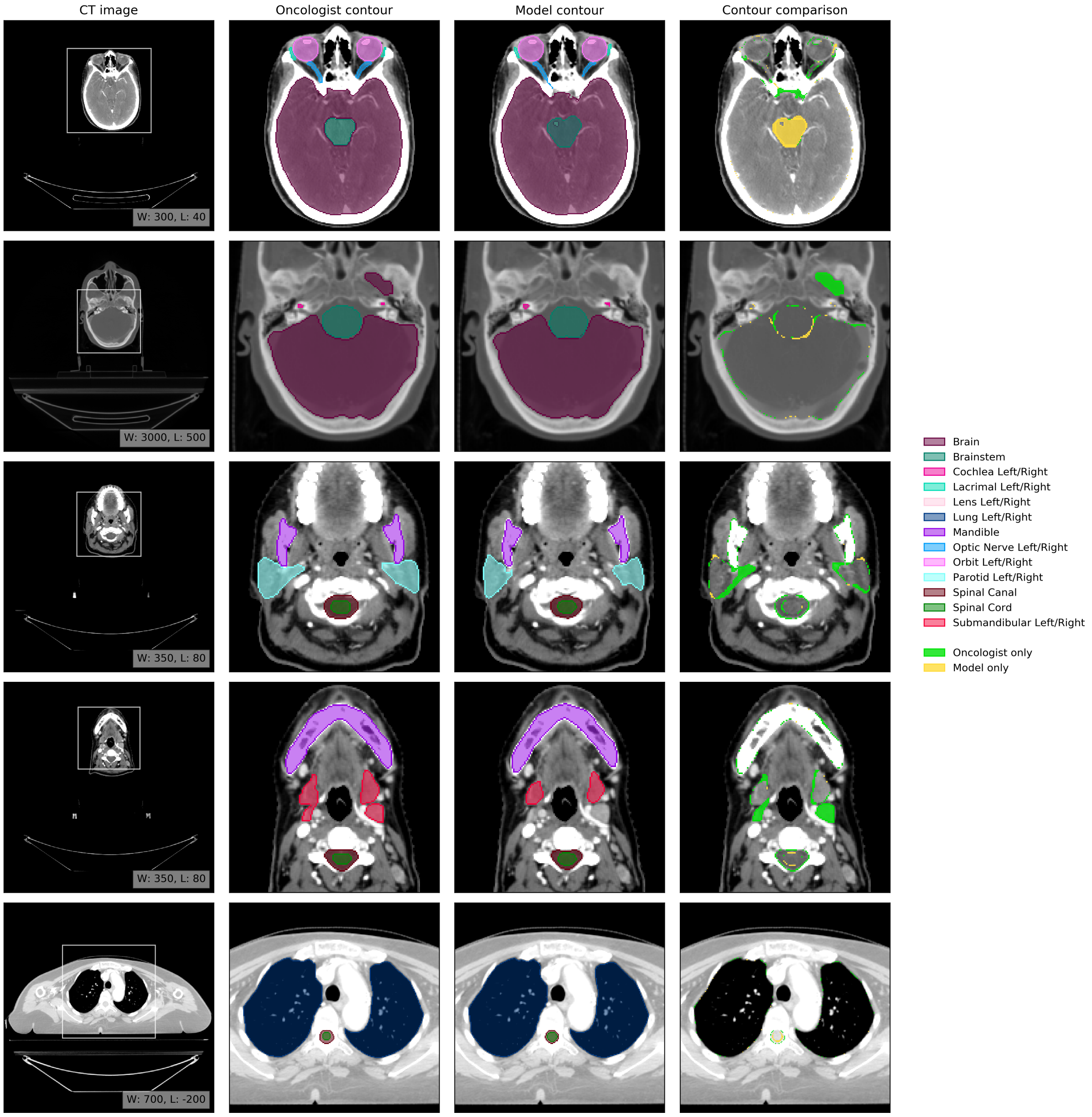

Figure S1 | Example results. Axial slices at five representative levels from the raw CT scan of 70-74 year old female patient from the UCLH test set. The levels shown as 2D slices have been selected to demonstrate all 21 OARs included in this study. The window levelling has been adjusted for each to best display the anatomy present. (**Oncologist contour**) The ground truth segmentation, as defined by experienced radiographers and arbitrated by a head and neck specialist oncologist. (**Model contour**) Segmentations produced by our model. (**Contour comparison**) Contoured by Oncologist only (green region) or Model only (yellow region). Best viewed on a display.

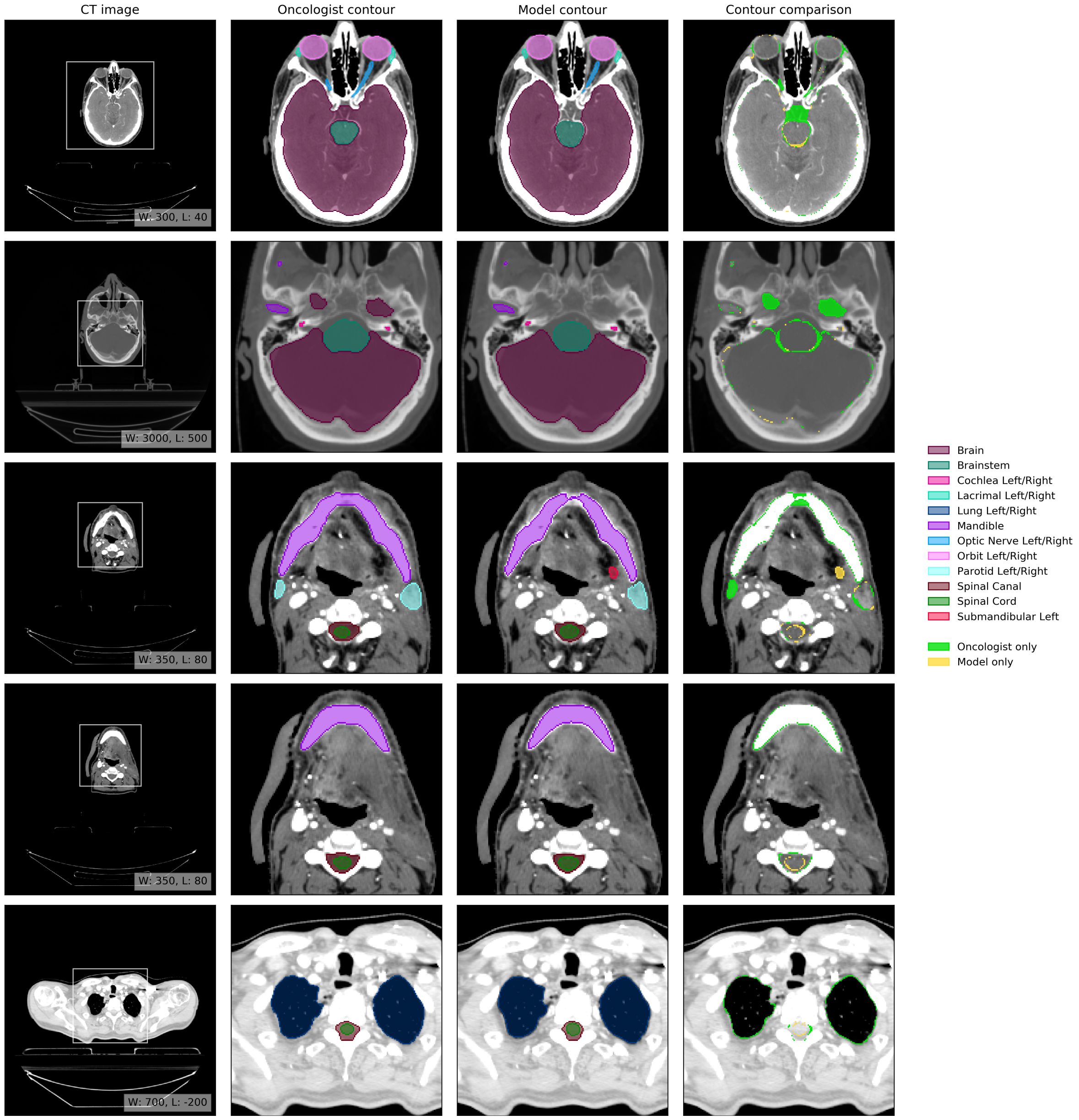

Figure S2 | Example results. Axial slices at five representative levels from the raw CT scan of 70-74 year old male patient from the UCLH test set. The levels shown as 2D slices have been selected to demonstrate all 21 OARs included in this study. The window levelling has been adjusted for each to best display the anatomy present. (**Oncologist contour**) The ground truth segmentation, as defined by experienced radiographers and arbitrated by a head and neck specialist oncologist. (**Model contour**) Segmentations produced by our model. (**Contour comparison**) Contoured by Oncologist only (green region) or Model only (yellow region). Best viewed on a display.

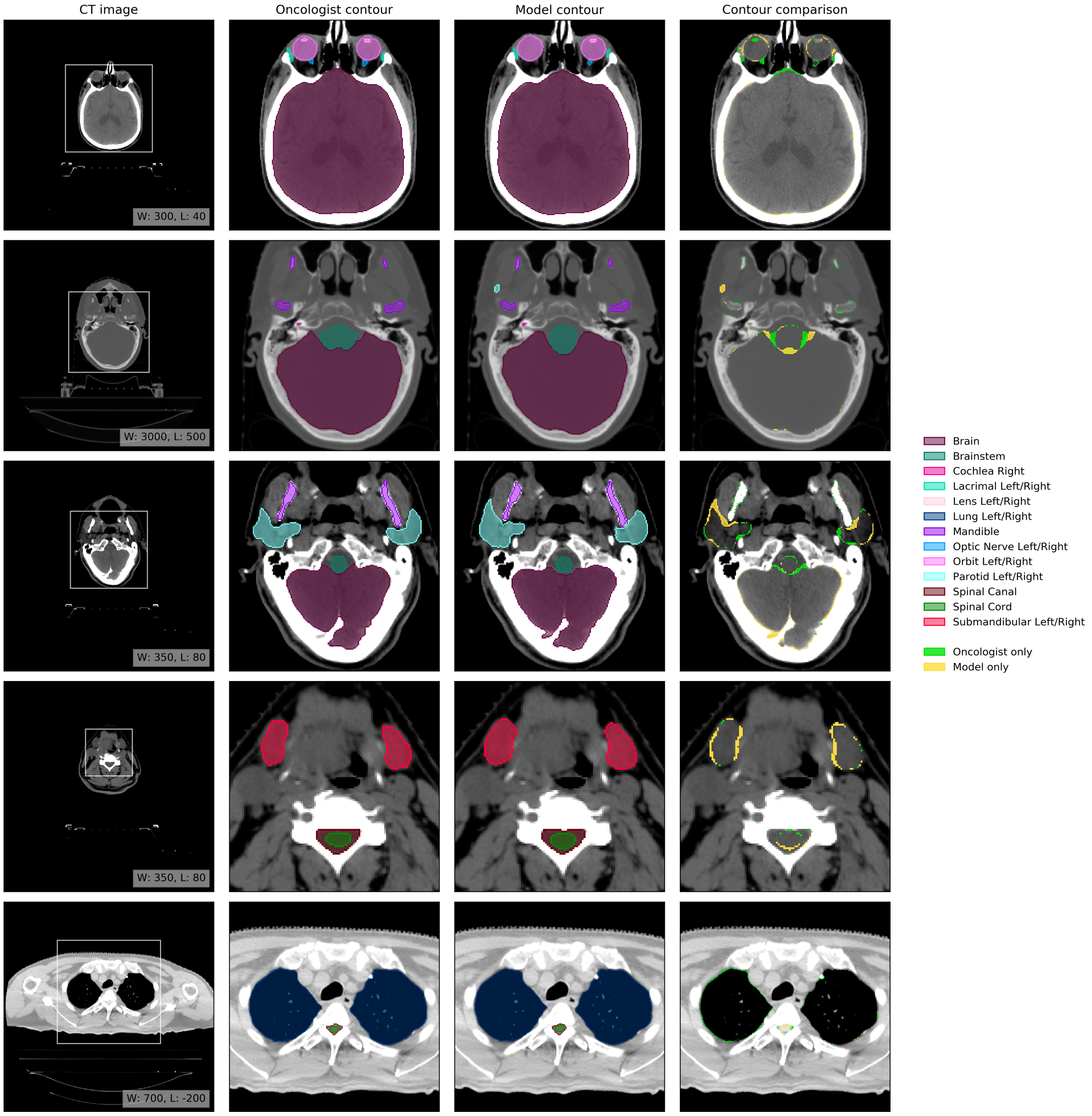

Figure S3 | Example results. (**a1-e1**) Axial slices at five representative levels from the raw CT scan of a 66 year old male patient with a right base of tongue cancer and bilateral lymph node involvement selected from the Head-Neck Cetuximab TCIA dataset (patient 0522c0057; [48]) were selected to best demonstrate the OARs included in the work. The levels shown as 2D slices have been selected to demonstrate all 21 OARs included in this study. The window levelling has been adjusted for each to best display the anatomy present. (**a2-e2**) The ground truth segmentation, as defined by experienced radiographers and arbitrated by a head and neck specialist oncologist. (**a3-e3**) Segmentations produced by our model. (**a4-e4**) Overlap between the model (yellow line) and the ground truth (blue line). Two further randomly selected TCIA set scans are shown in Figure S4 and Figure S5. Best viewed on a display.

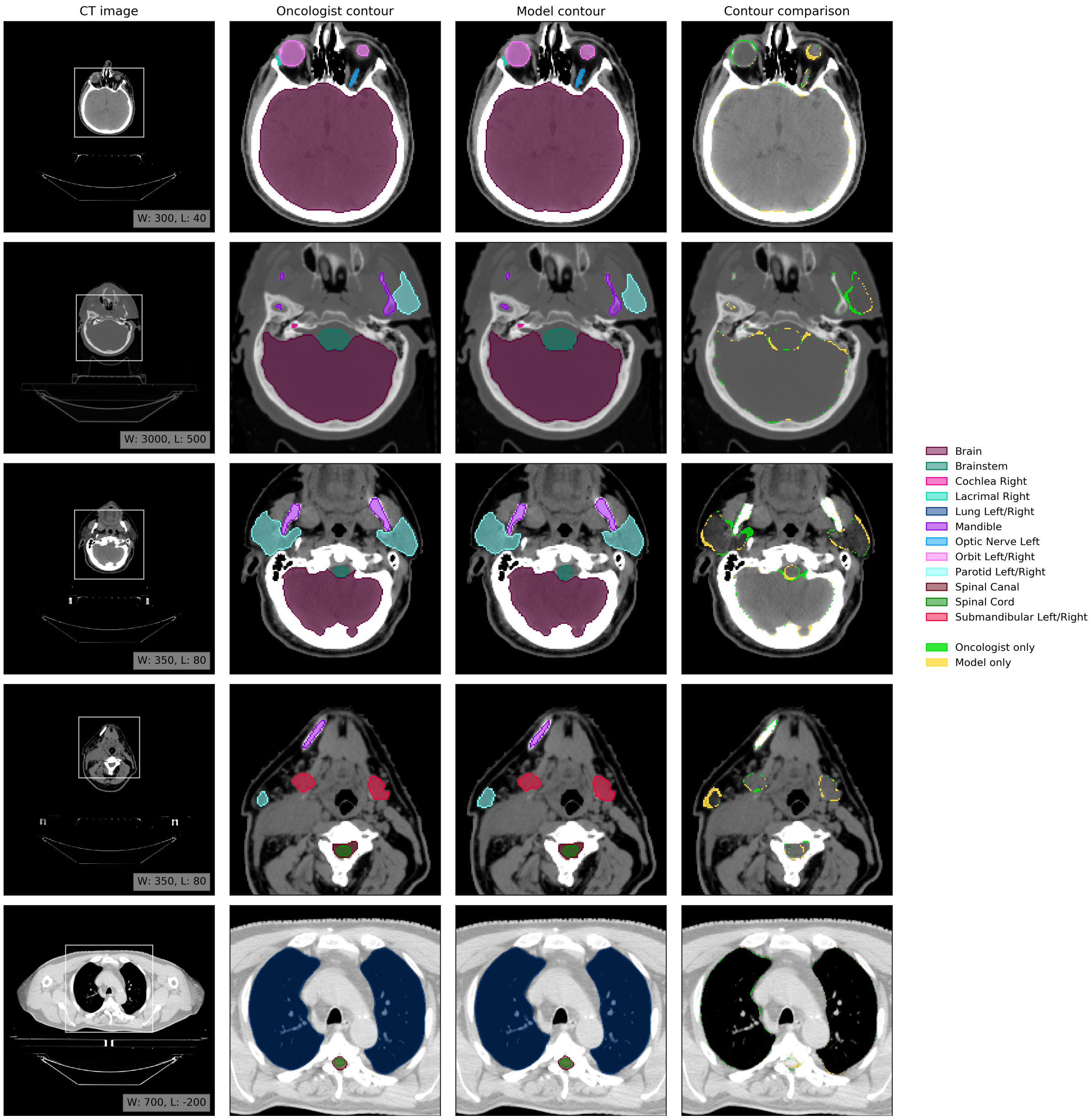

Figure S4 | Example results from a second randomly selected case from the TCIA test set. Five axial slices from the scan of a 58 year old male patient with a cancer of the right tonsil selected from the Head-Neck Cetuximab TCIA dataset (patient 0522c0416; [48]). (a1-e1) The raw CT scan slices at five representative levels were selected to best demonstrate the OARs included in the work. The window levelling has been adjusted for each to best display the anatomy present. (a2-e2) The ground truth segmentation was defined by experienced radiographers and arbitrated by a head and neck specialist oncologist. (a3-e3) The model produced segmentations of the same structures. Overlap between the model (yellow line) and the ground truth (blue line) is shown in (a4-e4). Best viewed on a display.

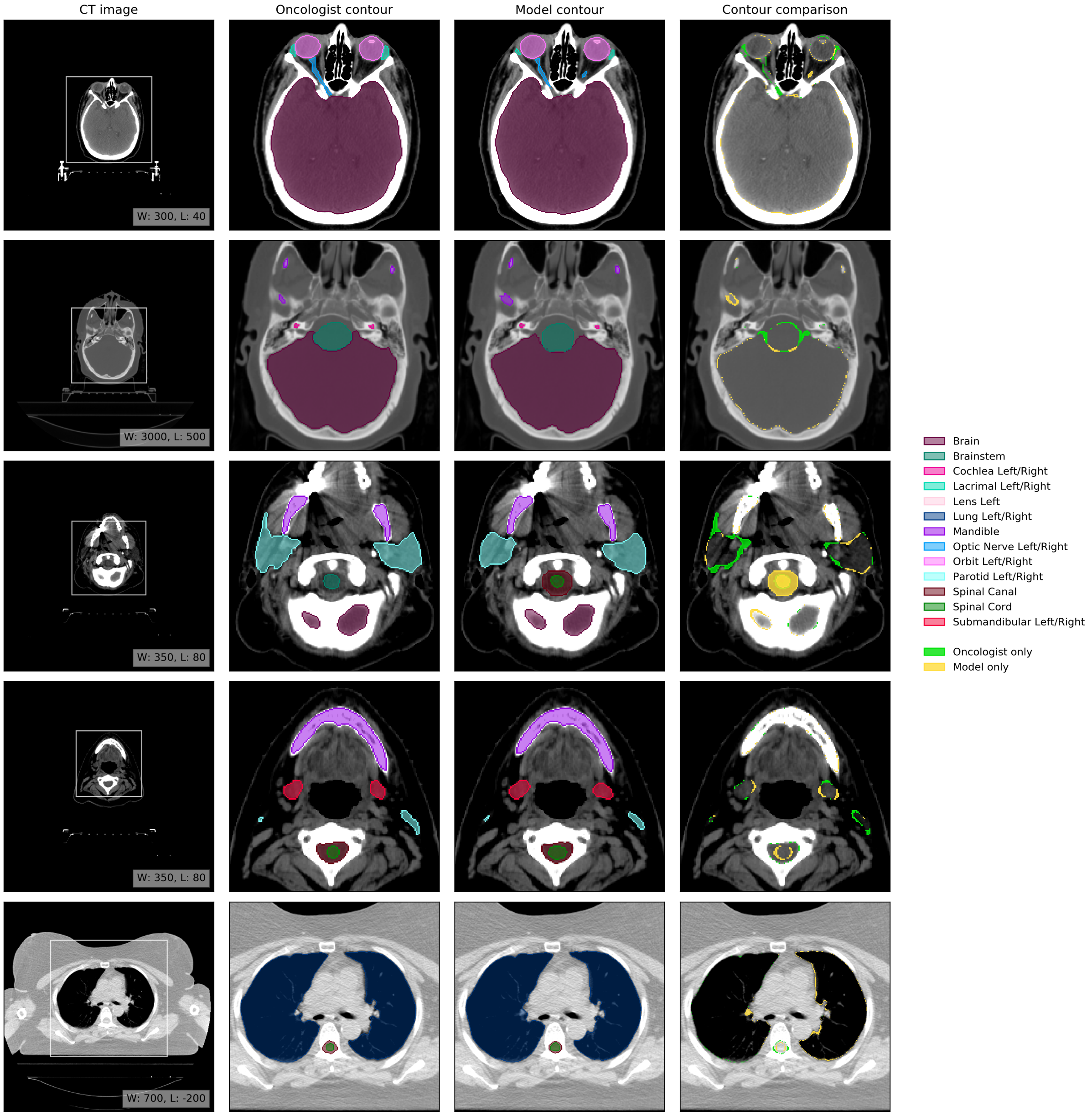

Figure S5 | Example results from a third randomly selected case from the TCIA test set. Five axial slices from the scan of a 53 year old female patient with a left oropharyngeal cancer with base of tongue invasion included selected from the Head-Neck Cetuximab TCIA dataset (patient 0522c0251; [48]). (a1-e1) The raw CT scan slices at five representative levels were selected to best demonstrate the OARs included in the work. The window levelling has been adjusted for each to best display the anatomy present. (a2-e2) The ground truth segmentation was defined by experienced radiographers and arbitrated by a head and neck specialist oncologist. (a3-e3) The model produced segmentations of the same structures. Overlap between the model (yellow line) and the ground truth (blue line) is shown in (a4-e4). Best viewed on a display.

Table S1 | Surface DSC on UCLH data set

Organ	M/H	UCLH-01	UCLH-02	UCLH-03	UCLH-04	UCLH-05	UCLH-06	UCLH-07	UCLH-08	UCLH-09	UCLH-10	UCLH-11	UCLH-12	UCLH-13	UCLH-14	UCLH-15	UCLH-16	UCLH-17	UCLH-18	UCLH-19	UCLH-20	UCLH-21	mean, stddev	diff.
		UCLH test set patient ID																						
**Brain**	(M)	98	97	94	95	94	96	96	94	94	96	95	95	94	96	94	97	97	95	93	97	96	95.5±1.3	-0.9
(81738.4 mm^2)	(H)	97	99	94	96	98	96	97	96	96	96	94	97	95	95	95	99	98	97	96	97	98	96.4±1.3	
**Brainstem**	(M)	100	99	84	97	99	98	99	99	97	99	98	98	99	98	98	97	99	99	98	97	100	97.7±3.2	0.7
(6555.3 mm^2)	(H)	98	99	95	95	98	98	96	97	97	97	95	98	95	91	97	99	99	99	100	95	99	97.0±2.1	
**Cochlea-Lt**	(M)	100	95	96	98	100	100	94	99	99	100	100	98	91	96	98	98	97	94	96	100	100	97.6±2.5	0.8
(83.8 mm^2)	(H)	100	100	100	89	100	100	100	99	100	98	86	100	98	97	100	80	99	97	99	89	100	96.7±5.5	
**Cochlea-Rt**	(M)	95	100	96	100	94	89	96	100	98	100	100	100	96	99	100	96	99	98	100	100	100	97.8±2.9	0.3
(84.5 mm^2)	(H)	100	100	96	93	100	93	100	99	100	98	93	100	100	100	100	90	95	100	94	94	100	97.4±3.2	
**Lacrimal-Lt**	(M)	99	100	97	99	99	95	97	99	96	93	99	100	98	90	99	98	95	96	98	100	99	97.4±2.5	0.8
(671.4 mm^2)	(H)	93	99	99	100	93	99	96	97	97	94	100	97	93	96	98	99	100	99	84	97	97	96.5±3.6	
**Lacrimal-Rt**	(M)	100	99	87	98	97	93	98	99	91	98	99	99	100	98	100	94	96	99	99	96	95	96.9±3.3	0.9
(658.2 mm^2)	(H)	100	99	95	98	92	83	96	91	93	98	100	96	100	99	99	96	96	99	98	91	98	96.0±4.0	
**Lens-Lt**	(M)	100	100	100	98	100	100	0	100	94	63	96	91	100	100	97	99	100	99	90	87	100	91.1±22.0	-3.0
(222.7 mm^2)	(H)	95	100	100	96	100	99	59	99	97	63	95	97	100	100	97	100	100	96	85	100	100	94.1±11.3	
**Lens-Rt**	(M)	100	100	100	99	100	100	0	95	98	100	100	95	97	99	98	100	100	96	100	93	94	93.5±21.0	-1.2
(218.4 mm^2)	(H)	100	100	100	96	100	100	40	96	100	94	96	96	99	99	94	97	100	97	100	100	90	94.8±12.6	
**Lung-Lt**	(M)	100	99	97	98	99	98	100	98	98	97	99	99	97	99	99	99	100	99	98	98	99	98.7±0.8	0.0
(44876.4 mm^2)	(H)	100	99	99	99	99	99	100	97	98	98	97	100	98	99	99	99	99	98	100	98	99	98.6±0.9	
**Lung-Rt**	(M)	99	99	95	98	99	98	98	98	95	99	99	99	97	99	99	99	100	99	99	98	98	98.3±1.3	-0.2
(45978.6 mm^2)	(H)	100	98	99	98	98	98	99	98	98	99	96	99	98	98	99	100	99	98	99	98	98	98.5±0.9	
**Mandible**	(M)	95	98	96	95	98	96	99	93	96	94	89	98	98	93	90	97	98	99	94	95	95	95.6±2.7	-2.4
(21268.1 mm^2)	(H)	95	98	99	100	97	98	100	96	98	100	94	97	99	99	97	100	97	98	97	97	99	97.9±1.5	
**Optic-Nerve-Lt**	(M)	98	97	97	98	99	98	97	98	97	95	100	100	99	93	97	97	100	97	97	95	98	97.5±1.6	0.7
(723.6 mm^2)	(H)	97	99	83	98	99	100	100	100	96	92	91	99	100	98	96	98	92	99	100	94	100	96.8±4.1	
**Optic-Nerve-Rt**	(M)	99	100	86	99	100	96	97	99	100	100	99	99	99	83	98	89	95	98	98	98	100	96.7±4.6	-0.4
(722.3 mm^2)	(H)	96	100	73	99	99	98	99	100	100	95	99	99	100	96	99	95	99	98	100	97	100	97.2±5.6	
**Orbit-Lt**	(M)	91	99	92	99	97	100	97	100	99	97	94	98	99	97	100	98	99	95	97	99	98	97.4±2.4	0.3
(2553.3 mm^2)	(H)	94	100	97	95	98	100	96	100	98	94	97	90	99	99	98	94	95	99	98	100	96	97.1±2.5	
**Orbit-Rt**	(M)	94	98	98	100	94	100	96	94	100	97	93	97	100	98	99	97	98	98	98	98	100	97.4±2.1	-0.2
(2547.3 mm^2)	(H)	96	99	98	98	95	100	92	100	99	97	95	96	100	97	99	98	95	98	100	99	99	97.6±2.0	
**Parotid-Lt**	(M)	93	90	97	98	92	84	89	95	98	96	95	97	91	96	86	95	95	82	89	97	87	92.4±4.7	-2.2
(7779.0 mm^2)	(H)	95	91	98	90	96	84	100	97	97	95	93	97	98	96	93	95	99	98	92	91	92	94.6±3.7	
**Parotid-Rt**	(M)	88	93	78	98	93	90	90	95	96	93	82	97	90	98	88	93	93	85	84	93	96	91.1±5.2	-3.3
(7714.8 mm^2)	(H)	95	96	99	97	97	89	96	96	98	94	87	89	97	100	84	98	95	93	97	96	91	94.4±4.1	
**Spinal-Canal**	(M)	93	96	89	95	93	97	95	94	92	96	95	88	93	95	98	94	93	93	93	93	98	93.8±2.4	0.4
(16014.9 mm^2)	(H)	99	97	91	89	98	97	96	91	96	93	96	88	89	96	93	92	94	93	89	88	97	93.4±3.5	
**Spinal-Cord**	(M)	99	100	100	100	100	99	100	100	97	100	99	100	99	100	98	100	98	99	100	99	100	99.4±0.8	-0.4
(7660.0 mm^2)	(H)	100	100	100	98	100	100	100	100	100	100	100	100	100	100	99	100	99	100	100	100	100	99.7±0.5	
**Submandibular-Lt**	(M)	67	74	79	83	-	98	-	87	86	83	90	93	87	89	90	89	95	94	97	94	99	88.1±8.0	-4.5
(3478.8 mm^2)	(H)	97	96	81	90	-	93	-	93	90	94	96	91	88	89	92	96	91	97	98	91	99	92.6±4.2	
**Submandibular-Rt**	(M)	77	97	84	92	-	95	-	93	96	-	-	95	90	96	85	89	100	98	94	94	99	92.4±5.8	-0.3
(3279.1 mm^2)	(H)	95	93	86	86	-	94	-	95	94	-	-	96	93	97	78	92	96	93	93	97	99	92.7±5.0	
aggr. surface DSC[1]	(M)	96.6	97.4	93.2	95.8	96.5	96.3	96.9	95.3	95.2	96.8	96.4	96.8	95.3	97.4	96.2	96.8	97.9	96.8	95.5	97.1	97.2		
	(H)	97.7	98.1	95.0	95.6	97.9	96.8	98.2	96.2	97.1	97.3	95.2	96.9	96.1	97.1	96.5	98.0	97.8	97.3	97.0	96.8	98.2		
difference		-1.1	-0.7	-1.8	0.2	-1.5	-0.6	-1.3	-0.9	-1.8	-0.5	1.1	-0.1	-0.8	0.3	-0.3	-1.2	0.0	-0.5	-1.5	0.3	-1.0		

Numbers below the organ name show the average surface area of this organ in the UCLH test set.
M: our model performance
H: human (radiographer) performance
Colours indicate the performance difference:

< -10%	(model is worse)
-10% to -5%	(model is slightly worse)
-5% – +5%	(model and human are on par)
+5% to +10%	(model is slightly better)
> +10%	(model is better)

Table S2 | Volumetric DSC on UCLH data set

Organ	M/H	UCLH-01	UCLH-02	UCLH-03	UCLH-04	UCLH-05	UCLH-06	UCLH-07	UCLH-08	UCLH-09	UCLH-10	UCLH-11	UCLH-12	UCLH-13	UCLH-14	UCLH-15	UCLH-16	UCLH-17	UCLH-18	UCLH-19	UCLH-20	UCLH-21	mean, stddev	diff.
**Brain**	(M)	99	99	99	99	99	99	99	99	99	99	99	99	99	99	99	99	99	99	99	99	99	99± 0	-0.1
(1316891.7 mm^3)	(H)	99	99	99	99	99	99	99	99	99	99	99	99	99	99	99	99	99	99	99	99	99	99.2±0.17	
**Brainstem**	(M)	93	93	83	91	92	90	93	90	89	89	91	89	91	90	89	91	92	92	90	91	94	91± 2	0.5
(26422.5 mm^3)	(H)	90	93	86	88	92	92	91	90	92	87	88	91	87	85	88	92	92	93	91	90	93	90.1±2.40	
**Cochlea-Lt**	(M)	94	82	71	72	98	84	73	80	67	81	81	72	68	81	88	80	82	82	83	95	81	81± 8	2.9
(62.4 mm^3)	(H)	84	92	65	52	94	87	90	69	69	82	59	89	68	81	89	44	83	87	82	73	98	77.9±13.97	
**Cochlea-Rt**	(M)	74	86	72	87	75	73	77	80	73	85	82	86	68	85	81	72	81	79	88	81	82	79± 6	-1.0
(61.3 mm^3)	(H)	84	88	73	68	88	80	80	71	79	88	68	95	77	91	84	52	83	96	83	73	88	80.3±10.14	
**Lacrimal-Lt**	(M)	77	82	66	76	73	74	79	75	68	68	81	67	68	60	72	69	71	73	75	80	79	73± 6	-1.1
(785.6 mm^3)	(H)	73	86	71	81	78	74	78	72	71	67	85	65	69	71	72	82	85	76	57	77	68	74.1±7.03	
**Lacrimal-Rt**	(M)	82	79	59	76	69	62	79	75	63	76	77	77	75	76	75	70	69	70	74	70	67	72± 6	0.6
(768.1 mm^3)	(H)	82	80	74	78	69	52	72	64	57	80	81	74	75	78	77	75	71	70	67	62	71	71.8±7.77	
**Lens-Lt**	(M)	81	86	87	82	89	89	0	88	79	56	84	76	88	83	89	76	87	85	79	68	81	78±19	-5.0
(244.1 mm^3)	(H)	83	87	83	87	93	84	27	83	85	58	86	84	91	90	85	88	86	93	84	91	89	82.7±14.16	
**Lens-Rt**	(M)	93	87	90	78	89	88	0	81	83	85	90	83	89	84	88	84	86	85	86	75	74	81±19	-3.3
(237.6 mm^3)	(H)	90	90	90	86	88	88	18	85	91	85	83	85	89	85	81	93	85	91	91	87	81	83.9±14.98	
**Lung-Lt**	(M)	99	98	93	97	99	99	99	98	98	99	99	98	98	99	99	99	99	99	99	99	99	98± 1	-0.1
(510340.2 mm^3)	(H)	99	99	95	97	99	99	99	99	99	99	98	99	99	99	99	99	98	98	99	99	99	98.6±0.89	
**Lung-Rt**	(M)	99	99	89	96	99	99	99	98	98	99	100	98	98	99	99	98	99	99	99	99	98	98± 2	-0.4
(561923.9 mm^3)	(H)	99	99	94	95	99	99	99	99	99	99	98	99	99	99	99	99	99	99	99	99	99	98.6±1.22	
**Mandible**	(M)	94	95	92	89	94	94	95	92	94	91	89	94	95	92	92	94	96	95	93	95	91	93± 2	-2.7
(67811.7 mm^3)	(H)	95	96	96	95	96	96	98	94	93	97	94	96	97	97	97	97	95	97	96	96	96	95.8±1.23	
**Optic-Nerve-Lt**	(M)	68	77	75	81	80	80	80	78	75	72	78	80	84	75	62	76	80	78	81	79	81	77± 5	-3.1
(781.3 mm^3)	(H)	81	80	67	77	83	83	84	83	78	67	77	87	86	79	82	76	80	84	82	80	86	80.3±5.22	
**Optic-Nerve-Rt**	(M)	78	83	63	83	77	71	79	78	82	85	73	79	77	70	57	70	78	70	70	69	81	75± 7	-4.4
(792.4 mm^3)	(H)	76	80	52	83	82	81	82	84	84	70	78	80	87	76	78	75	81	84	89	82	84	79.4±7.40	
**Orbit-Lt**	(M)	93	96	92	96	95	95	95	95	96	92	95	94	95	96	95	95	96	94	92	95	93	95± 1	0.6
(9813.0 mm^3)	(H)	92	96	94	94	94	94	94	96	95	92	95	91	94	95	94	92	94	95	91	96	94	93.9±1.41	
**Orbit-Rt**	(M)	94	96	94	96	95	95	95	93	96	93	93	95	95	95	94	94	95	95	93	96	95	95± 1	0.5
(9906.5 mm^3)	(H)	94	95	94	95	93	95	93	96	95	93	94	94	94	94	93	94	93	94	93	96	95	94.2±0.90	
**Parotid-Lt**	(M)	83	82	89	89	84	80	83	87	91	88	88	89	86	85	82	88	88	78	81	88	77	85± 4	-3.1
(27542.6 mm^3)	(H)	87	85	91	87	88	83	92	91	92	89	87	88	91	88	87	89	92	90	85	84	85	88.1±2.75	
**Parotid-Rt**	(M)	82	86	75	89	85	85	83	89	91	88	81	89	83	89	78	87	86	77	77	84	83	84± 5	-3.4
(27663.6 mm^3)	(H)	88	88	89	89	89	85	88	90	93	90	85	82	90	91	78	90	89	86	89	86	84	87.5±3.35	
**Spinal-Canal**	(M)	91	94	93	94	92	95	94	91	90	94	94	91	93	94	95	91	93	92	92	93	95	93± 1	-0.3
(56388.6 mm^3)	(H)	96	95	92	92	96	94	95	94	95	92	93	89	90	94	93	93	93	94	89	92	95	93.1±1.98	
**Spinal-Cord**	(M)	84	82	88	89	76	70	88	85	68	78	74	86	70	70	68	82	58	84	86	64	87	78± 9	-3.6
(15607.7 mm^3)	(H)	90	90	81	78	85	82	84	86	78	84	84	88	83	84	82	85	66	68	83	74	78	81.6±6.00	
**Submandibular-Lt**	(M)	60	68	68	80	–	90	–	82	84	82	83	88	82	84	82	86	89	90	91	88	92	83± 8	-4.8
(10197.2 mm^3)	(H)	89	88	74	85	–	87	–	88	88	90	90	85	84	86	85	92	88	91	92	88	92	87.5±3.97	
**Submandibular-Rt**	(M)	75	90	77	84	–	88	–	87	91	–	–	86	84	89	79	85	95	90	89	87	87	86± 5	-0.8
(9295.9 mm^3)	(H)	86	87	78	83	–	89	–	89	89	–	–	85	88	90	76	88	91	87	89	90	90	86.8±4.02	

The patient-ID columns UCLH-01 to UCLH-21 are grouped under the heading "UCLH test set patient ID".

Numbers below the organ name show the average volume of this organ in the UCLH test set.
M: our model performance
H: human (radiographer) performance

Colors indicate performance differences: green: model is better, red: model is worse

Table S3 | Number of labelled scans in UCLH test set

		Brain	Brainstem	Cochlea		Lacrimal		Lens		Lung		Mandible	Optic Nerve		Orbit		Parotid		Spinal-Canal	Spinal-Cord	Submandibular	
				lt	rt	lt	rt	lt	rt	lt	rt		lt	rt	lt	rt	lt	rt			lt	rt
Number of scans		75	45	8	8	75	73	75	73	71	72	74	17	15	19	16	33	32	23	24	64	65
Dense segmentation				✓	✓	✓	✓	✓	✓				✓	✓								
Number of labelled slices	axial	309	225							265	275	300			95	75	165	160	345	350	250	260
	coronal	374	225							355	360	375			95	80	165	160	0	0	320	325
	sagittal	374	225							355	360	375			95	80	165	160	0	0	320	325

Table S4 | Surface DSC on TCIA data set

TCIA test set patient ID

Organ	M/H	0522c 0017	0522c 0057	0522c 0161	0522c 0226	0522c 0248	0522c 0251	0522c 0331	0522c 0416	0522c 0419	0522c 0427	0522c 0457	0522c 0479	0522c 0629	0522c 0659	0522c 0667	0522c 0669	0522c 0708	0522c 0768	0522c 0770	0522c 0773	0522c 0845	TCGA-CV-7236	TCGA-CV-7243	TCGA-CV-7245	TCGA-CV-A6JO	TCGA-CV-A6JY	TCGA-CV-A6K0	TCGA-CV-A6K1	mean, stddev	diff.
**Brain** (84054.9 mm^2)	(M)	95	94	96	94	96	94	96	98	94	71	98	95	97	94	96	97	97	98	91	94	97	97	97	97	97	96	96	94	94.9±4.8	-1.3
	(H)	97	96	97	95	93	96	96	97	95	96	98	96	97	97	97	96	96	98	96	97	97	98	96	96	97	96	95	95	96.3±1.0	
**Brainstem** (6531.0 mm^2)	(M)	84	72	92	79	86	65	92	94	87	62	99	93	97	99	99	100	99	98	72	82	95	97	98	87	98	96	94	94	89.5±10.5	-7.5
	(H)	96	97	88	96	93	99	94	97	97	93	100	96	97	100	100	99	98	100	97	97	99	98	98	98	99	98	98	98	97.1±2.5	
**Cochlea-Lt** (93.3 mm^2)	(M)	98	99	97	99	100	100	100	100	97	96	95	100	100	85	84	98	97	100	100	100	92	100	100	100	98	100	95	100	97.6±4.1	2.4
	(H)	100	100	90	94	94	91	100	90	100	94	100	99	94	85	83	87	97	100	99	100	92	93	91	100	100	100	92	100	95.2±5.1	
**Cochlea-Rt** (85.5 mm^2)	(M)	100	99	99	97	100	100	97	100	94	95	98	93	100	98	100	100	98	100	99	100	100	100	88	99	95	100	100	100	98.2±2.7	8.6
	(H)	100	100	0	100	95	88	90	100	100	94	100	100	100	99	8	100	95	100	98	95	83	93	77	95	100	100	100	99	89.6±24.5	
**Lacrimal-Lt** (535.1 mm^2)	(M)	(99)	(92)	(97)	(89)	(73)	(86)	(97)	(97)	(83)	(87)	(98)	(81)	(93)	(97)	(96)	(99)	(98)	(99)	(96)	(98)	(100)	(99)	(98)	(100)	(98)	(100)	(96)	(98)	94.4±6.6	-0.1
	(H)	(100)	(91)	(85)	(98)	(88)	(100)	(92)	(90)	(86)	(100)	(100)	(95)	(100)	(98)	(99)	(99)	(99)	(95)	(91)	(88)	(93)	(93)	(89)	(96)	(98)	(94)	(95)	(98)	94.6±4.7	
**Lacrimal-Rt** (553.9 mm^2)	(M)	(97)	(99)	(99)	(92)	(75)	(86)	(88)	(87)	(96)	(82)	(89)	(96)	(88)	(99)	(95)	(100)	(85)	(97)	(100)	(98)	(90)	(98)	(100)	(96)	(91)	(97)	(93)	(92)	93.1±6.1	-0.2
	(H)	(100)	(98)	(96)	(98)	(91)	(95)	(84)	(82)	(97)	(99)	(100)	(88)	(96)	(98)	(99)	(100)	(83)	(96)	(94)	(94)	(89)	(99)	(93)	(92)	(93)	(82)	(93)	(83)	93.3±5.8	
**Lens-Lt** (193.5 mm^2)	(M)	100	96	93	100	96	100	100	94	100	100	100	0	100	93	95	100	100	94	95	97	97	100	98	99	96	99	94	100	94.1±18.3	-4.2
	(H)	100	96	100	95	100	100	100	100	96	100	100	89	100	100	96	100	100	100	96	97	95	100	98	100	99	97	100	99	98.3±2.6	
**Lens-Rt** (193.4 mm^2)	(M)	100	0	95	100	100	100	93	0	100	96	100	72	90	92	100	94	100	96	100	96	100	92	96	100	100	100	94	99	89.4±25.4	-7.2
	(H)	100	74	97	100	100	96	96	81	99	100	100	85	100	97	100	100	100	96	100	98	100	93	96	100	100	99	100	100	96.7±6.2	
**Lung-Lt** (56292.2 mm^2)	(M)	(100)	(99)	(100)	(99)	(98)	(99)	(92)	(97)	(99)	(99)	(99)	(97)	(94)	(97)	(99)	(100)	(99)	(99)	(99)	(98)	(98)	(99)	(99)	(99)	(99)	(100)	(99)	(99)	98.4±1.6	0.2
	(H)	(99)	(99)	(100)	(97)	(97)	(96)	(97)	(95)	(99)	(98)	(100)	(98)	(98)	(100)	(100)	(100)	(100)	(99)	(100)	(97)	(98)	(99)	(99)	(99)	(99)	(96)	(100)	(91)	98.2±1.9	
**Lung-Rt** (58043.6 mm^2)	(M)	(99)	(99)	(92)	(99)	(98)	(99)	(90)	(95)	(99)	(99)	(99)	(97)	(93)	(99)	(98)	(100)	(99)	(99)	(99)	(95)	(99)	(99)	(99)	(98)	(99)	(99)	(100)	(97)	97.8±2.4	-0.5
	(H)	(99)	(99)	(100)	(97)	(98)	(99)	(97)	(95)	(98)	(98)	(100)	(98)	(97)	(100)	(99)	(100)	(100)	(99)	(99)	(95)	(99)	(99)	(99)	(98)	(98)	(97)	(100)	(96)	98.3±1.4	
**Mandible** (20867.9 mm^2)	(M)	97	95	95	94	98	98	93	99	96	75	100	91	99	99	98	93	100	98	82	95	98	99	98	99	91	96	95	99	95.4±5.3	-2.6
	(H)	99	100	96	97	100	94	98	99	96	99	100	99	100	98	98	97	100	99	99	94	92	98	97	98	99	99	99	98	98.0±2.0	
**Optic-Nerve-Lt** (717.6 mm^2)	(M)	92	100	98	99	98	95	98	91	95	99	100	99	93	98	95	100	93	96	94	100	97	99	97	98	100	99	99	95	97.0±2.6	0.9
	(H)	89	99	100	99	91	99	99	95	86	90	98	99	96	99	96	98	94	96	91	100	100	100	100	92	95	96	98	95	96.1±3.8	
**Optic-Nerve-Rt** (719.9 mm^2)	(M)	89	99	97	97	99	92	99	96	98	99	95	100	89	99	93	88	96	100	99	100	96	100	97	95	100	99	99	92	96.4±3.5	-0.7
	(H)	88	99	95	97	95	100	98	99	95	100	99	99	95	100	97	97	99	99	97	100	99	100	95	93	96	95	98	96	97.2±2.7	
**Orbit-Lt** (2320.5 mm^2)	(M)	93	99	93	99	95	86	96	94	98	99	100	96	99	99	99	97	99	94	94	86	97	92	90	89	95	91	92	95	94.9±3.8	-1.0
	(H)	97	98	95	92	94	93	95	96	98	95	98	99	98	95	98	100	100	97	98	90	100	94	89	89	99	96	94	96	95.9±3.1	
**Orbit-Rt** (2360.3 mm^2)	(M)	96	93	94	97	97	93	92	91	98	95	100	96	96	98	100	99	99	93	93	91	99	91	95	96	96	88	98	94	95.3±3.0	-0.5
	(H)	100	94	92	95	98	95	98	97	95	97	96	93	100	100	98	99	100	95	95	91	96	93	97	89	99	91	96	91	95.7±3.0	
**Parotid-Lt** (7991.9 mm^2)	(M)	91	82	91	96	92	92	77	95	75	95	97	95	95	95	95	93	99	97	87	89	95	94	95	94	85	67	97	97	91.1±7.5	-3.3
	(H)	94	82	93	94	96	98	88	91	99	96	98	93	97	95	97	97	97	97	99	89	90	93	97	94	97	90	97	97	94.4±3.9	
**Parotid-Rt** (8322.3 mm^2)	(M)	96	91	89	93	97	84	93	90	68	94	97	93	91	90	97	95	98	96	90	95	93	91	92	95	69	95	90	93	91.2±7.0	-3.5
	(H)	96	90	91	89	98	95	93	98	92	95	99	99	93	96	98	98	97	97	94	95	95	94	97	92	92	97	90	95	94.8±2.8	
**Spinal-Canal** (18036.4 mm^2)	(M)	(93)	(92)	(93)	(91)	(92)	(89)	(87)	(98)	(94)	(87)	(93)	(91)	(91)	(98)	(92)	(86)	(92)	(91)	(88)	(94)	(88)	(96)	(87)	(93)	(93)	(91)	(93)	(96)	91.8±3.1	-2.9
	(H)	(95)	(96)	(96)	(91)	(94)	(92)	(96)	(95)	(96)	(97)	(99)	(94)	(95)	(96)	(92)	(91)	(94)	(95)	(97)	(98)	(93)	(97)	(95)	(93)	(93)	(94)	(96)	(92)	94.7±2.0	
**Spinal-Cord** (8623.7 mm^2)	(M)	99	99	99	99	100	99	100	100	94	98	100	98	99	100	100	100	100	98	99	100	99	100	99	100	100	100	98	100	99.1±1.1	-0.6
	(H)	99	100	100	100	99	100	100	100	100	100	100	99	99	100	100	100	100	99	100	100	100	100	100	100	100	100	100	100	99.8±0.3	
**Submandibular-Lt** (3167.6 mm^2)	(M)	(75)	(86)	(89)	(88)	(97)	(96)	(66)	(96)	(99)	(83)	(94)	(89)	(86)	(98)	(80)	(83)	(91)	(93)	(72)	(98)	(89)	(-)	83	(59)	(-)	(-)	(88)	(89)	86.7±9.9	-3.7
	(H)	(91)	(98)	(98)	(94)	(98)	(97)	(77)	(98)	(98)	(96)	(100)	(90)	(96)	(99)	(96)	(99)	(96)	(97)	(94)	(100)	(69)	(-)	97	(0)	(-)	(-)	(91)	(91)	90.4±19.7	
**Submandibular-Rt** (3156.2 mm^2)	(M)	(67)	(83)	(65)	(82)	(93)	(95)	(86)	(95)	(97)	(95)	(95)	(91)	(96)	(97)	(96)	(90)	(92)	(92)	(93)	(83)	(93)	(33)	(36)	(73)	(-)	(89)	(-)	(67)	83.6±17.1	1.6
	(H)	(83)	(99)	(91)	(84)	(92)	(98)	(89)	(96)	(91)	(100)	(100)	(90)	(92)	(100)	(89)	(100)	(97)	(96)	(99)	(97)	(87)	(0)	(0)	(77)	(-)	(86)	(-)	(0)	82.0±30.2	
aggr. surface DSC[1]	(M)	95.2	93.0	95.0	94.1	96.0	92.7	94.9	97.0	91.3	77.2	98.5	94.7	96.8	95.5	96.7	96.5	97.9	97.5	89.2	94.0	96.9	97.1	96.3	96.5	93.4	94.5	95.4	95.2		
	(H)	97.1	95.9	95.6	95.1	95.2	96.5	95.9	97.1	95.4	96.6	98.5	96.6	97.3	97.3	97.4	96.8	97.1	98.3	96.8	96.2	95.8	97.2	96.3	96.2	97.4	96.1	96.1	96.1		
difference		-1.8	-2.9	-0.6	-1.0	0.8	-3.7	-0.9	-0.1	-4.1	-19.4	-0.0	-1.9	-0.4	-1.7	-0.6	-0.3	0.8	-0.8	-7.6	-2.2	1.1	-0.2	-0.0	0.3	-4.0	-1.6	-0.7	-0.9		

Numbers below the organ name show the average surface area of this organ in the test set.
M: our model performance
H: human (radiographer) performance
numbers in brackets indicate that this organ for this patient would not be segmented in current clinical practise
[1]: aggregated only over organs that would be segmented for this patient in current clinical practise. I.e. numbers in brackets were excluded.
Colours indicate the performance difference:

< -10%	(model is worse)
-10% to -5%	(model is slightly worse)
-5% – +5%	(model and human are on par)
+5% to +10%	(model is slightly better)
> +10%	(model is better)

Table S5 | Volumetric DSC on TCIA data set

		TCIA test set patient ID																													
Organ	M/H	0522c 0017	0522c 0057	0522c 0161	0522c 0226	0522c 0248	0522c 0251	0522c 0331	0522c 0416	0522c 0419	0522c 0427	0522c 0457	0522c 0479	0522c 0629	0522c 0659	0522c 0667	0522c 0669	0522c 0708	0522c 0768	0522c 0770	0522c 0773	0522c 0845	TCGA-CV-7236	TCGA-CV-7243	TCGA-CV-7245	TCGA-CV-A6JO	TCGA-CV-A6JY	TCGA-CV-A6K0	TCGA-CV-A6K1	mean, stddev	diff.
**Brain** (1386616.3 mm^3)	(M)	99	99	99	99	99	99	99	99	99	93	99	99	99	99	99	99	99	99	99	99	99	99	99	99	99	99	99	99	99± 1	-0.3
	(H)	99	99	99	99	99	99	99	99	99	99	100	99	99	99	99	99	99	99	99	99	99	99	99	99	99	99	99	99	99.1±0.24	
**Brainstem** (27236.8 mm^3)	(M)	82	73	87	77	83	73	85	90	82	65	93	84	90	92	93	94	90	91	73	81	88	90	89	85	89	90	91	85	85± 7	-4.9
	(H)	90	90	87	86	86	92	87	89	89	87	95	87	91	95	95	91	91	93	89	92	91	89	90	89	91	87	89	89	90.0±2.53	
**Cochlea-Lt** (70.9 mm^3)	(M)	75	80	84	92	81	81	83	87	77	80	91	89	89	65	58	81	74	77	85	89	85	79	70	90	75	96	66	76	80± 9	5.6
	(H)	79	74	76	82	74	70	71	69	89	72	100	76	76	48	50	60	67	91	77	82	70	79	63	88	79	81	72	80	74.9±10.93	
**Cochlea-Rt** (66.3 mm^3)	(M)	84	90	93	88	89	83	75	87	75	73	81	81	77	68	77	81	76	89	80	81	85	84	58	86	77	87	84	79	81± 7	11.4
	(H)	71	92	0	82	62	60	51	87	97	76	100	84	72	68	0	80	74	88	74	62	55	67	52	73	93	73	84	72	69.6±23.12	
**Lacrimal-Lt** (599.8 mm^3)	(M)	72	57	76	54	36	55	47	73	57	49	75	35	69	60	72	70	74	69	54	66	72	73	72	81	70	78	70	66	64±12	-2.9
	(H)	83	53	52	68	58	80	48	63	60	75	89	56	71	81	80	74	74	62	63	50	74	67	66	58	76	67	70	65	67.3±10.44	
**Lacrimal-Rt** (633.8 mm^3)	(M)	60	64	80	59	36	56	50	58	66	51	60	58	59	71	68	79	59	69	69	70	65	70	78	66	63	69	66	66	64± 9	-4.0
	(H)	79	68	69	66	55	76	52	62	65	77	99	55	75	81	80	85	56	63	59	67	68	79	61	58	70	53	65	57	67.8±11.02	
**Lens-Lt** (200.2 mm^3)	(M)	89	85	75	89	87	94	88	75	86	86	84	0	92	75	80	91	77	80	76	86	79	93	85	87	84	87	84	91	82±17	-6.1
	(H)	90	81	96	89	94	91	90	88	84	80	100	54	93	95	92	94	82	86	86	87	80	94	87	88	90	87	89	88	87.7±8.04	
**Lens-Rt** (201.1 mm^3)	(M)	90	0	65	89	88	91	78	0	89	85	95	27	79	71	76	72	88	85	80	79	90	79	83	91	89	87	80	94	76±25	-8.7
	(H)	90	44	87	89	96	90	83	46	85	92	100	42	90	90	93	90	92	83	92	90	82	84	83	87	96	84	91	93	84.5±14.72	
**Lung-Lt** (656382.6 mm^3)	(M)	99	99	99	99	99	99	98	99	99	99	99	99	97	98	98	98	98	99	99	99	98	99	99	99	99	99	99	99	99± 1	0.0
	(H)	99	99	99	98	99	98	98	98	99	99	100	99	98	99	99	99	99	99	99	98	98	99	99	99	99	98	99	97	98.7±0.66	
**Lung-Rt** (722139.3 mm^3)	(M)	99	99	98	99	99	99	97	99	99	99	99	99	97	98	98	98	98	99	99	99	99	99	99	99	99	99	99	99	99± 1	-0.1
	(H)	99	99	99	98	99	99	99	98	99	99	100	99	98	99	99	99	99	99	99	98	99	99	99	99	99	98	99	98	98.9±0.47	
**Mandible** (59383.2 mm^3)	(M)	92	94	92	92	96	95	91	94	95	79	96	89	94	96	94	93	96	91	88	92	94	95	93	95	90	95	95	96	93± 4	-1.3
	(H)	96	97	93	94	94	90	92	95	92	95	100	96	94	96	96	95	96	92	95	90	90	95	95	92	94	95	94	94	94.2±2.21	
**Optic-Nerve-Lt** (757.1 mm^3)	(M)	64	80	78	77	82	70	72	69	83	76	79	80	81	78	72	80	73	77	72	83	82	79	79	83	84	84	84	80	78± 5	-1.4
	(H)	67	80	76	80	74	89	80	75	77	71	83	83	81	85	80	84	81	79	70	83	83	83	81	78	81	84	81	73	79.3±4.86	
**Optic-Nerve-Rt** (747.0 mm^3)	(M)	69	73	78	77	79	68	72	71	81	81	79	82	71	74	70	71	80	69	79	86	82	84	80	74	78	81	85	63	76± 6	-2.0
	(H)	73	82	77	74	69	82	75	69	77	77	97	81	76	83	86	83	80	73	67	78	87	81	70	76	80	81	84	78	78.4±6.25	
**Orbit-Lt** (8520.3 mm^3)	(M)	90	94	92	95	93	90	94	92	95	94	94	93	95	96	95	95	93	90	92	88	93	92	91	91	93	91	92	92	93± 2	-0.7
	(H)	93	94	93	91	92	93	92	93	93	94	96	95	92	94	96	96	96	93	95	87	95	92	92	90	95	95	92	94	93.3±2.05	
**Orbit-Rt** (8706.5 mm^3)	(M)	93	93	92	94	95	93	92	90	95	94	94	94	94	96	96	95	96	91	92	89	94	91	94	91	93	91	94	93	93± 2	-0.3
	(H)	95	92	91	93	94	93	93	94	93	96	95	91	95	96	96	94	96	92	94	88	93	92	94	90	94	93	93	92	93.4±1.85	
**Parotid-Lt** (29887.7 mm^3)	(M)	81	78	82	88	82	85	72	88	75	88	88	86	86	86	89	84	89	87	85	82	88	84	89	88	83	64	90	90	84± 6	-3.0
	(H)	83	79	83	86	86	90	84	85	90	90	96	83	88	90	89	89	90	88	91	85	86	84	90	87	89	83	90	88	87.1±3.37	
**Parotid-Rt** (31237.3 mm^3)	(M)	85	84	86	89	87	80	84	86	69	87	91	86	82	82	90	86	89	86	85	88	86	83	83	86	76	85	87	84	85± 4	-2.7
	(H)	84	85	86	85	88	88	83	90	85	89	98	88	82	89	90	90	91	88	89	88	87	85	89	84	88	86	85	86	87.4±3.08	
**Spinal-Canal** (63887.1 mm^3)	(M)	92	91	94	93	92	90	91	94	92	89	92	92	91	94	90	88	90	91	90	93	89	94	91	91	94	92	92	94	92± 2	-2.3
	(H)	93	95	94	92	94	93	95	90	94	95	99	94	93	95	94	93	95	94	95	94	92	96	96	91	94	92	95	92	93.9±1.77	
**Spinal-Cord** (19029.9 mm^3)	(M)	75	83	64	86	88	81	85	84	57	78	89	76	86	88	90	83	79	79	82	88	86	68	80	70	78	83	83	80	80± 8	-4.0
	(H)	81	86	75	85	87	85	85	83	70	83	92	83	85	87	88	88	80	80	87	90	88	79	85	78	85	88	87	88	84.3±4.63	
**Submandibular-Lt** (9339.5 mm^3)	(M)	67	82	80	83	90	88	62	88	93	82	90	83	81	91	79	78	88	88	68	91	85	–	82	60	–	–	84	83	82± 9	-2.8
	(H)	81	91	89	86	91	89	74	90	93	91	100	83	90	93	92	92	91	92	88	94	70	–	90	0	–	–	85	85	84.7±18.32	
**Submandibular-Rt** (9226.6 mm^3)	(M)	59	78	61	78	86	88	82	89	92	89	90	85	89	88	88	82	86	87	86	82	88	19	25	78	–	83	–	65	78±18	0.3
	(H)	78	91	86	78	86	91	86	89	89	93	100	83	87	93	87	95	92	89	91	91	82	0	0	78	–	79	–	0	77.5±28.49	

Numbers below the organ name show the average volume of this organ in the test set.
M: our model performance
H: human (radiographer) performance
Colors indicate performance differences: green: model is better, red: model is worse

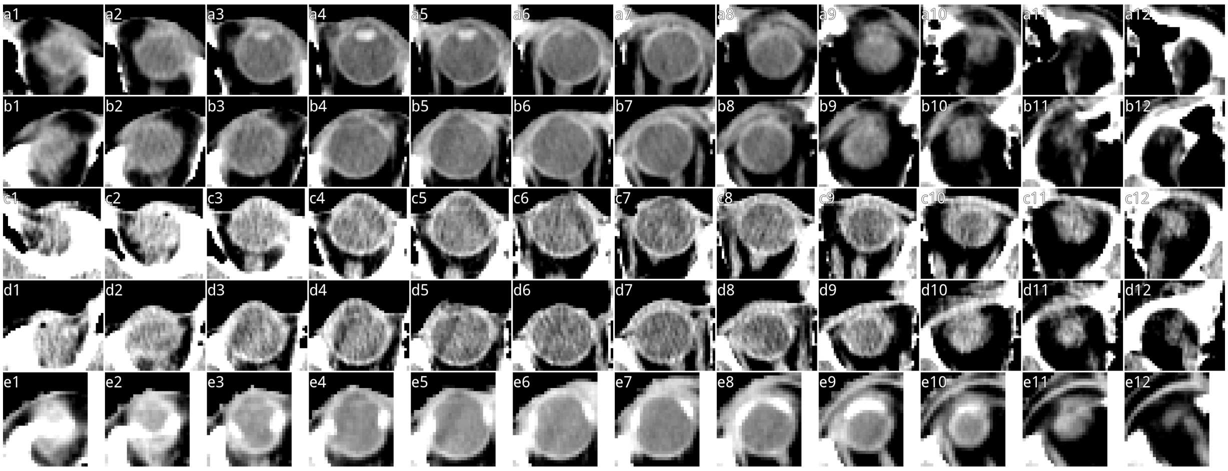

Figure S6 │ Missed lens predictions across the TCIA test set. Consecutive axial slices of eyes showing both a typical lens and the four cases where the model predictions omitted the lens. The window level is at a constant W:140 L:0. (**a1-a12**) 12 slices through a single eye in which the model was able to detect the lens, which is clearly visible in (a3-a6). (a1) is the upper most slice, (a12) the lower most. (**b1-e12**) Similar to the first row, but these four cases are those for which the model was unable to differentiate the lens from the rest of the eye. Note that all four cases are considerably more challenging than for the first row.

Table S6 | Volumetric DSC performance of our model and previously published results. An overview of previously published automatic segmentation works that reported volumetric DSC for the OARs included in this study on planning head and neck CT scans. The datasets and ground truths used varied between studies making comparison difficult. Despite this, we show results alongside our evaluation of our model, radiographers and oncologists against our ground truth across multiple datasets. The latter assesses inter-observer variation between oncologists.

Study	Method	Brain	Brainstem	Cochlea		Lacrimal		Lens		Lung		Mandible	Optic Nerve		Orbit		Parotid		Spinal Canal	Spinal Cord	Submandibular	
				lt	rt	lt	rt	lt	rt	lt	rt		lt	rt	lt	rt	lt	rt			lt	rt
van Dijk (2020) [61]	CNN		83[2]									95[2]					84[2]	83[2]		87[2]	77[2]	78[2]
Zhong (2019) [31]	CNN												89									
Močnik (2018) [29]	CNN																92					
Ren (2018) [30]	CNN												72	70			77					
Ibragimov (2017) [28]	CNN											90	64	65	88	88	77	78		87	70	73
Fritscher (2016) [27]	CNN																81				65	
Guo (2020) [46]	FCN		88									94	72	71			87	86			78	81
Qiu (2020) [59]	FCN											95										
Sun (2020) [60]	FCN		86									94			90	90	84	81		89	78	77
Wong (2020) [62]	FCN		83										47				80			79	82	
Liang (2020) [58]	FCN		92					88	87			94	74		93	93	88			90	81	
Xue (2019) [69]	FCN		90									96	86	84			89	89			86	85
Chan (2019) [63]	FCN		89									91					85	86		87	84	85
Gao (2019) [64]	FCN		86					81	79				64	62	88	91	77	80		87		
Lei (2019) [66]	FCN		87										66				86					
Sun (2019) [67]	FCN							85	84				80	82	94	94						
Jiang (2019) [65]	FCN		88									93					85	86			79	77
van Rooij (2019) [45]	FCN		64														83	83			82	81
Tang (2019) [44]	FCN		86					82	83			93	75	76	92	92	85	85		86	81	83
Rhee (2019) [43]	FCN	98	86	65	68			73	70			87	89	90	89	90	83	83		83		
Tappeiner (2019) [42]	FCN		82									91	64	63			80	81				
Men (2019) [41]	FCN		90									92					86	86				
Wang (2019) [40]	FCN		88									93	74	74			86	85			76	73
Nikolov (2018) [70]	FCN	99	88	65	75	69	70	81	80	99	99	96	76	77	95	95	85	85	95	88	85	85
Kodym (2018) [39]	FCN		92									95	80				90				88	
Tong (2018) [36]	FCN		87									94	65	69			84	83			76	81
Zhu (2018) [35]	FCN		87									93	72	71			88	87			81	81
Willems (2018) [38]	FCN		92	75	73							96					86	90			79	88
Hänsch (2018) [34]	FCN																86					
Liang (2018) [37]	FCN		90					83	84			91	66	72			85	85				
Tong (2019) [68]	GAN		87									94	66	70			85	86			81	82
Gacha (2018) [24]	HAS											80										
Raudashl (2017) [25]	HAS		88									93	62				84				78	
Fritcher (2014) [71]	HAS		87[2,3]														84[2,3]	83[2,3]				
Walker (2014) [23]	HAS		97	56								98	71				89			90	73	
Thomson (2014) [22]	HAS			30[2,3]													79[3]				80[3]	
Fortunati (2013) [20]	HAS		78					67					62		81					85		
Qazi (2011) [72]	HAS		91									93										
Wu (2019) [26]	Machine learning											89					74	75			73	73
Tam (2018) [73]	Machine learning		91	67	72			75	74			85			94	94	83	82		83	87	87
Wang (2018) [56]	Machine learning		90									94					82	83				
Torosdagli (2017) [57]	Machine learning											94										
Wang (2019) [74]	Multi-ABAS		84									75	56	53			75	74			74	72
Ayyalusamy (2019) [75]	Multi-ABAS		83[2]									85[2]					81[2]			84[2]		
Haq (2019) [76]	Multi-ABAS		76									85					76	76		84	60	60
McCarroll (2018) [77]	Multi-ABAS	98	81	47						48		84			85		78			71		
Liu (2016) [78]	Multi-ABAS		92									90					87	85		80	83	80
Hoang Duc (2015) [21]	Multi-ABAS		82[2,3]												68[2,3]		70[2,3]	71[2,3]		79[2,3]		
Tao (2015) [79]	Multi-ABAS		86	43	42														77			
Wachinger (2015) [80]	Multi-ABAS																78[2,3]	77[2,3]				
Zhu (2013) [81]	Multi-ABAS	95[2]	72[2]									90[2]					72[2]			72[2]	70[2]	
Teguh (2011) [82]	Multi-ABAS		78[1]														79			78[1]	70	
Han (2011) [83]	Multi-ABAS																85	86				
Sims (2009) [84]	Multi-ABAS		77									82					84	86				
Sims (2009) [84]	Multi-ABAS		58									78					69	66				
Han (2008) [85]	Multi-ABAS		84[2,3]									91[2,3]					83[2,3]			75[2,3]	70[2,3]	
Hoogeman (2008) [86]	Multi-ABAS		71[1]																	71[1]		
Huang (2019) [87]	Single-ABAS											84	73	71			84	84		82	75	78
Daisne (2013) [19]	Single-ABAS		75[2]														72[2]					
Hardcastle (2012) [88]	Single-ABAS		86[2]														80[2]	80[2]		83[2]		
La Macchia (2012) [89]	Single-ABAS		81	69	63							86					78	79	81			
Zhang (2007) [90]	Single-ABAS		80[2]									85[2]					81[2]	80[2]		83[2]		
Radiographer (TCIA) (28 scans)	Manual	99.1 ±0.2	90.0 ±2.5	74.9 ±10.9	69.6 ±23.1	67.3 ±10.4	67.8 ±11.0	87.7 ±8.0	84.5 ±14.7	98.7 ±0.7	98.9 ±0.5	94.2 ±2.2	79.3 ±4.9	78.4 ±6.2	93.3 ±2.1	93.4 ±1.9	87.1 ±3.4	87.4 ±3.1	93.9 ±1.8	84.3 ±4.6	84.7 ±18.3	77.5 ±28.5
Our model (TCIA) (28 scans)	Deep Learning	98.8 ±1.1	85.1 ±7.1	80.5 ±8.8	81.0 ±7.2	64.4 ±11.9	63.8 ±9.0	81.6 ±16.6	75.7 ±24.5	98.7 ±0.6	98.8 ±0.7	92.9 ±3.5	77.9 ±5.0	76.3 ±5.8	92.6 ±2.0	93.1 ±1.8	84.1 ±5.8	84.6 ±4.2	91.7 ±1.6	80.3 ±7.6	81.8 ±8.7	77.8 ±18.1
Radiographer (UCLH) (21 scans)	Manual	99.2 ±0.2	90.1 ±2.4	77.9 ±14.0	80.3 ±10.1	74.1 ±7.0	71.8 ±7.8	82.7 ±22.6	83.9 ±23.8	98.6 ±0.9	98.6 ±1.3	95.8 ±1.2	80.3 ±5.2	79.4 ±7.4	93.9 ±1.4	94.2 ±0.9	88.1 ±2.8	87.5 ±3.4	93.1 ±2.0	81.6 ±6.0	87.5 ±4.0	86.8 ±4.0
Our model (UCLH) (21 scans)	Deep Learning	99 ±0.2	91 ±2.2	81 ±8.2	79 ±5.7	73 ±5.6	72 ±5.8	78 ±25.0	81 ±25.8	98 ±1.3	98 ±2.2	931 ±1.9	77 ±4.8	75 ±7.0	95 ±1.3	95 ±1.0	85 ±3.8	84 ±4.5	93 ±1.4	78 ±8.9	83 ±8.4	86 ±4.9
Oncologist (UCLH) (8 - 75 scans)[4]	Manual	99.0[5]	91.9[5]	68.5 ±14.8	75.8 ±8.5	63.3 ±13.1	61.6 ±14.3	86.2 ±10.1	87.6 ±9.9	98.4[5]	98.6[5]	95.4[5]	77.1 ±6.3	76.0 ±7.1	94.8[5]	94.8[5]	90.1[5]	90.7[5]	94.9[5]	87.7[5]	91.1[5]	90.1[5]
Our model (PDDCA) (15 scans)	Deep Learning		84.2 ±5.2									93.8 ±1.9	71.6 ±6.2	69.1 ±5.9			88.1 ±2.0	86.6 ±3.5			76.5 ±9.1	79.2 ±6.5

Values for volumetric DCS are mean (± standard deviation) unless otherwise stated. "ABAS": atlas based auto segmentation. "CNN": convolutional neural network. "FCN": fully convolutional network. "GAN": generative adversarial network. "HAS": hybrid atlas-based segmentation.

[1] merged brainstem and spinal cord. [2] Values estimated from figures; actual values not reported. [3] Median; mean not reported. [4] Number of scans per organ varies, see Table S3. [5] Volumetric DSC estimated from sparse labels.

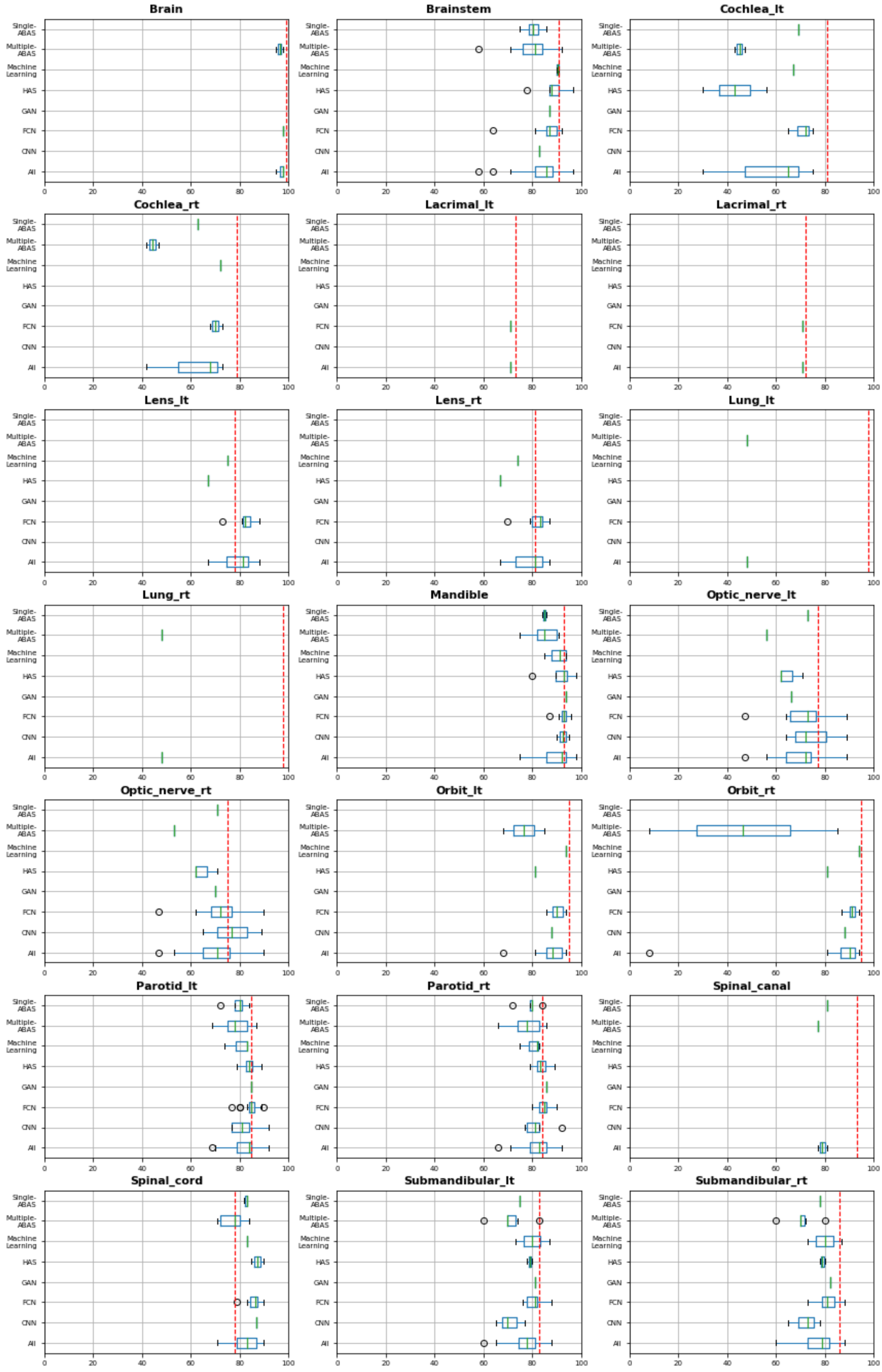

Figure S7 │ Comparison of volumetric DSC performance or our model and previously published results. The volumetric-DSC performance distribution is shown for each OAR. The performance distribution is shown for each method family and for all methods collectively. The blue boxes indicate the 1st and 3rd quartiles around the median (marked in green). The whiskers indicate most extreme, non-outlier data points. The red vertical lines indicate the performance of our model on the UCLH data.

Table S7 | Surface DSC on PDDCA data set

Organ	0522c 0555	0522c 0576	0522c 0598	0522c 0659	0522c 0661	0522c 0667	0522c 0669	0522c 0708	0522c 0727 c	0522c 0746	0522c 0788	0522c 0806	0522c 0845	0522c 0857	0522c 0878	mean, stddev
	PDDCA test set patient ID															
	off-site test set										on-site test set					
**Brainstem** (5042.8 mm^2)	84.4	85.4	87.4	89.9	98.4	79.4	98.9	99.9	98.1	72.3	98.2	89.7	91.2	95.0	71.5	89.3±9.0
**Mandible** (17215.2 mm^2)	96.1	98.5	97.9	97.5	96.6	98.4	96.4	99.8	98.6	98.3	97.0	93.7	97.6	96.2	94.1	97.1±1.6
**Optic-Nerve-Lt** (524.6 mm^2)	95.5	99.2	95.7	88.3	86.0	92.3	99.9	94.4	86.6	89.0	95.6	82.4	98.7	98.3	91.5	92.9±5.2
**Optic-Nerve-Rt** (480.7 mm^2)	95.0	95.6	95.5	93.2	89.6	93.2	95.2	96.0	96.3	79.8	97.2	83.7	96.7	97.0	91.3	93.0±4.9
**Parotid-Lt** (6710.1 mm^2)	96.4	96.6	99.1	95.7	97.5	95.5	97.4	99.2	89.8	95.1	98.6	92.1	98.1	98.6	96.6	96.4±2.5
**Parotid-Rt** (6630.9 mm^2)	93.2	94.0	97.7	91.3	98.2	98.1	96.7	96.0	93.8	74.5	97.1	93.2	98.4	97.4	85.5	93.7±6.1
**Submandibular-Lt** (2258.0 mm^2)	64.2	60.5	85.9	80.9	87.8	76.0	89.2	84.8	97.0	61.3	98.0	77.4	74.0	95.9	80.2	80.9±11.8
**Submandibular-Rt** (2296.7 mm^2)	81.2	73.4	93.6	85.3	85.2	92.9	86.9	85.8	99.7	68.0	98.9	85.5	79.6	78.0	80.7	85.0±8.5
aggr. surface dice	92.3	91.1	95.8	93.4	95.7	93.5	96.1	97.2	96.2	86.0	97.6	91.4	94.7	95.8	88.9	

Numbers below the organ name show the average surface area of this organ in the PDDCA test set.
Colours indicate the performance difference:

< -10%	(model is worse)
-10% to -5%	(model is slightly worse)
-5% – +5%	(model and human are on par)
+5% to +10%	(model is slightly better)
> +10%	(model is better)

Table S8 | Volumetric DSC on PDDCA data set

Organ	PDDCA test set patient ID															mean, stddev
	off-site test set										on-site test set					
	0522c 0555	0522c 0576	0522c 0598	0522c 0659	0522c 0661	0522c 0667	0522c 0669	0522c 0708	0522c 0727 c	0522c 0746	0522c 0788	0522c 0806	0522c 0845	0522c 0857	0522c 0878	
**Brainstem** (19778.8 mm^3)	82.0	82.7	83.0	84.9	88.8	76.4	88.5	92.8	86.7	76.3	89.9	85.3	86.5	85.5	73.7	84.2±5.2
**Mandible** (44477.1 mm^3)	94.2	92.1	95.8	94.9	90.4	94.9	94.6	96.3	96.0	95.4	92.8	90.8	92.5	94.7	91.6	93.8±1.9
**Optic-Nerve-Lt** (449.1 mm^3)	71.2	85.2	70.4	66.8	64.7	72.6	79.6	64.0	71.3	64.3	70.2	64.3	73.7	76.6	78.9	71.6±6.2
**Optic-Nerve-Rt** (384.3 mm^3)	69.8	68.6	75.6	63.1	61.9	69.3	62.7	73.8	78.7	63.0	69.1	62.5	65.4	80.5	72.9	69.1±5.9
**Parotid-Lt** (23677.4 mm^3)	87.9	89.0	91.1	85.6	90.1	89.2	88.7	88.7	84.0	87.0	90.1	84.6	88.9	88.8	87.8	88.1±2.0
**Parotid-Rt** (23828.3 mm^3)	87.2	88.1	90.8	82.4	89.4	90.2	87.0	86.9	87.1	76.8	87.6	86.5	88.8	88.0	82.6	86.6±3.5
**Submandibular-Lt** (5522.9 mm^3)	66.1	60.9	81.1	76.0	82.8	76.9	82.0	79.5	87.2	60.6	89.2	75.1	66.8	88.8	74.3	76.5±9.1
**Submandibular-Rt** (5660.5 mm^3)	80.6	75.8	83.8	76.6	76.7	86.8	83.1	77.2	89.6	66.7	89.8	82.4	74.9	72.5	71.6	79.2±6.5

Numbers below the organ name show the average volume of this organ in the PDDCA test set.
Colours indicate the performance difference:

< -10%	(model is worse)
-10% to -5%	(model is slightly worse)
-5% – +5%	(model and human are on par)
+5% to +10%	(model is slightly better)
> +10%	(model is better)
